# Long disordered regions of the C-terminal domain of Abelson tyrosine kinase have specific and additive functions in regulation and axon localization

**DOI:** 10.1371/journal.pone.0189338

**Published:** 2017-12-12

**Authors:** Han S. J. Cheong, Mark F. A. VanBerkum

**Affiliations:** Department of Biological Sciences, Wayne State University, Detroit, United States of America; University of Dublin Trinity College, IRELAND

## Abstract

Abelson tyrosine kinase (Abl) is a key regulator of actin-related morphogenetic processes including axon guidance, where it functions downstream of several guidance receptors. While the long C-terminal domain (CTD) of Abl is required for function, its role is poorly understood. Here, a battery of mutants of *Drosophila* Abl was created that systematically deleted large segments of the CTD from Abl or added them back to the N-terminus alone. The functionality of these Abl transgenes was assessed through rescue of axon guidance defects and adult lethality in *Abl* loss-of-function, as well as through gain-of-function effects in sensitized *slit* or *frazzled* backgrounds that perturb midline guidance in the *Drosophila* embryonic nerve cord. Two regions of the CTD play important and distinct roles, but additive effects for other regions were also detected. The first quarter of the CTD, including a conserved PxxP motif and its surrounding sequence, regulates Abl function while the third quarter localizes Abl to axons. These regions feature long stretches of intrinsically disordered sequence typically found in hub proteins and are associated with diverse protein-protein interactions. Thus, the CTD of Abl appears to use these disordered regions to establish a variety of different signaling complexes required during formation of axon tracts.

## Introduction

Abelson tyrosine kinase (Abl) is an essential protein that is a key regulator of cytoskeletal dynamics. Abl is known to aid in the regulation of actin dynamics underlying a myriad of cell processes, ranging from vesicle trafficking and Golgi architecture to cell motility and development [1–4]. Yet, understanding how Abl functions in these cellular processes remains challenging. During development in *Drosophila*, Abl participates in the processes of dorsal closure [5, 6], ventral furrow formation [7], cell adhesion [8, 9], photoreceptor morphogenesis [10, 11], and axon guidance. In particular, Abl has a long-appreciated role in nervous system development, and is thought to be a key link between axon guidance receptors and cytoskeletal activity underlying axon outgrowth and steering.

In this role, Abl functions downstream of a variety of receptors in both repulsive and attractive axon guidance pathways [12–17]. To carry out these functions, Abl cooperates with the adaptor protein Disabled to regulate the actin-associated proteins Enabled (Ena) and Abelson interacting protein (Abi), and further regulates the Rac GTPase through the Trio GEF [18]. Zygotic loss of function of *Abl* causes defects in midline crossing over of axons in the embryonic nerve cord [19] as well as stalling in the ISNb motoneurons at the periphery [20–23]. Indeed, the ladder-like structure of the embryonic nerve cord is severely disrupted if both zygotic and maternal contributions of Abl are removed [5]. To facilitate axon guidance at the midline, Abl is recruited by the Roundabout (Robo) and Frazzled (Fra) receptors, which govern the repulsive (Slit) and attractive (Netrin) pathways respectively. Abl also interacts with the Down Syndrome Cell Adhesion Molecule (Dscam) family of netrin receptors, where it may modulate Robo activity [17, 24, 25]. In the Robo-Slit pathway, Abl is physically recruited by Robo and phosphorylates it, and acts in concert with the actin-binding protein Capulet and microtubule-binding protein Orbit/MAST to regulate the growth cone cytoskeleton [12, 19, 26]. The Netrin receptor Frazzled also recruits Abl, although the precise role of Abl in this pathway is not well understood [13, 15, 27]. Indeed, perturbation of Abl levels in *fra*, *robo* or *slit* mutants cause a variety of axon guidance phenotypes that suggest that the role of Abl in these pathways is mechanistically complex [12–15, 27]. To begin to understand how Abl participates in these processes, several labs have previously focused on examining conserved elements within *Drosophila* Abl.

Initial work clearly documents the presence of both kinase-dependent and -independent functions of Abl in axon guidance and rescue of *Abl* mutant lethality [28]. The identification of significant kinase-independent activity suggests that Abl may function as a scaffold protein, helping form critical signaling complexes during these developmental processes. In this regard, the long CTD of Abl is a primary suspect for a scaffolding role. The possession of a long CTD in Abl family kinases is unique among non-receptor tyrosine kinases, and in *Drosophila* this region of roughly 1100 amino acids is indispensable for Abl function [28]. Comparison of the CTD sequence between vertebrates and invertebrates reveals little amino acid similarity, although all end with an F-actin binding domain (FABD). In vertebrates, this FABD participates in bundling of F-actin [29], localization of Abl to actin filaments [30], and regulation of Abl kinase activity [31, 32]. In *Drosophila*, the FABD is differentially required for axon guidance in various subsets of neurons; it is required for axon guidance of some commissural neurons, but dispensable in ISNb motoneurons [27]. Unique to *Drosophila* Abl are two putative EVH1-binding motifs (F/LPPPP) not present in the vertebrate Abl CTD, that are hypothesized to interact with the actin regulator Enabled (Ena) [33]. In the fly, Ena interacts both physically and genetically with Abl, and is also phosphorylated by Abl to suppress its activity [33–36]. Abl also regulates the subcellular distribution of Ena, and ectopic localization of Ena is a significant contributor to lethality in *Abl* loss-of-function [7, 37]. Thus, the EVH1-binding motifs were expected to be important for the function of Abl.

In an attempt to dissect the role of the CTD, previous work from the Peifer lab identified and examined four regions within the CTD that are conserved between insects (CR1-4) [6]. However, all but one of these regions proved to be dispensable for Abl’s function in a variety of actin-related morphogenetic processes. The dispensable regions include CR4, which contains the EVH1-binding motifs. Only deletion of CR1 significantly impairs the function of Abl in dorsal closure and axon guidance. This region contains a single ‘canonical’ PxxP motif, PAPPKR (a.a. 769–774 in Uniprot accession number# M9PFS1, consensus motifs, R/KxxPxxP or PxxPxR/K, as defined in [38, 39]) that aligns with one of three PxxP motifs in the CTD of vertebrate Abl homologs. In these vertebrate homologs, these three PxxP motifs in the CTD are known to be involved in the recruitment of the adaptor proteins Crk and Nck, as well as the Abelson-interacting (Abi) family of actin-regulating proteins [40–45]. Thus, a similar role in protein recruitment was hypothesized for the PxxP motif within the CR1 region [6]. Interestingly, several other canonical PxxP motifs exist within the *Drosophila* CTD, although their potential contribution to Abl function remains unknown.

In short, while the long CTD is essential, only small regions exhibit sequence conservation, and even these are not necessarily required for Abl function. So how might the CTD function? Here, we systematically deleted relatively large portions (here termed quarters) of the CTD from Abl and monitored their function. Conversely, we added these selected regions back to the N-terminal region of Abl, to determine which regions are by themselves sufficient to restore function to the kinase-active half of the molecule. Using this strategy, we note additive effects on Abl function throughout the long CTD, as well as two specific functions. We define a new region in the CTD (third quarter, or 3Q) that functions in the recruitment of Abl to axon tracts where it is important for axon guidance. Furthermore, we identify the first quarter (1Q) as a region critical to Abl activity, and while this region contains the previously-identified CR1 region, the conserved PxxP motif, while important, is not the sole contributor to Abl activity within this region. Finally we provide evidence that these regions are characterized by stretches of disorder, likely to function in protein-protein interactions typical of a hub protein. Future work will need to look at these regions in more detail, keeping in mind that specific sequence elements may be less important than overall structure.

## Results

**Fig 1:**
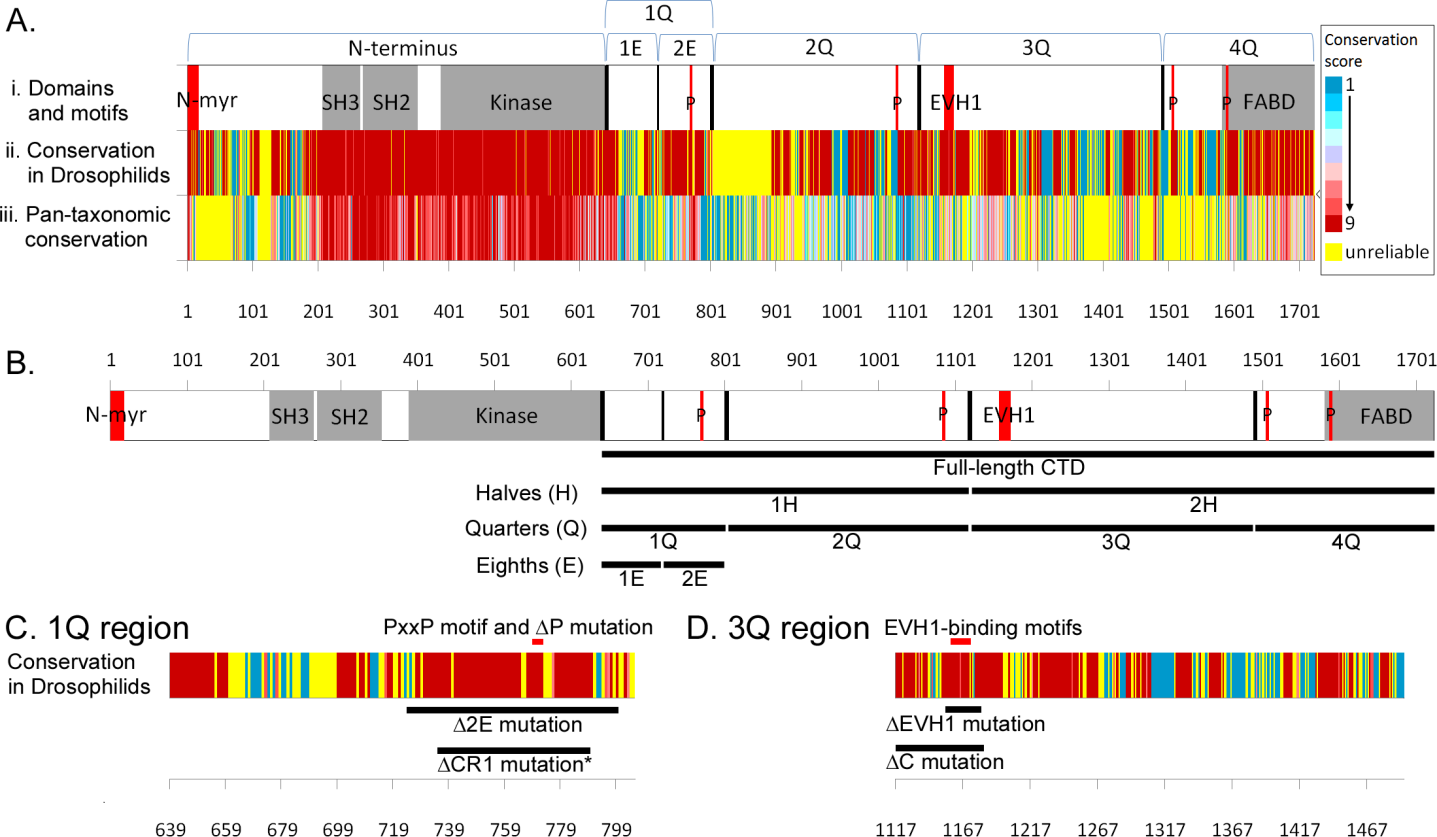
Abl structure and conservation, and CTD mutants made in this study. (A) The *Drosophila* Abl Abl CTD is relatively poorly conserved. (i) Presence of domains annotated by SMART (grey) and motifs (red) in *Drosophila* Abl. Notable motifs are N-terminal myristoylation site (N-myr), PxxP (P) and EVH1-binding motifs (EVH1). (ii) Conservation of amino acids of Abl in 11 Drosophilid species, as aligned by Clustal Omega and graded using ConSurf. ‘Unreliable’ conservation scores (yellow) have either less than 6 non-gapped residues in the alignment or a confidence interval of equal to or larger than 4 color grades. (iii) Conservation of amino acids using the same methodology as lane 2, but using 40 Abl homologs from both vertebrates and invertebrates. (B) Line cartoon of Drosophila Abl showing the large regions of the CTD (black lines) mutated in this paper. The CTD was divided into halves and quarters. The first quarter was further divided into first and second eighths. All transgenes carry a C-terminal FLAG-tag (not shown). (C) Close-up view of conservation of amino acids in the 1Q region of the Abl CTD, with conservation grade colors as in (A). Also shown are deletions removing the better-conserved second eighth (2E) region, and a comparison to the CR1 deletion as carried out in Rogers et al., 2016 (*). Deletion of the conserved PxxP motif (ΔP) is shown in lane 4. (D) Close-up view of conservation of amino acids in the 3Q region of the Abl CTD Also shown are a deletion removing the EVH1-binding motifs (ΔEVH1), and a deletion removing a highly conserved region of 3Q that includes EVH1 (ΔC).

The C-terminal domain of *Drosophila* Abl spans ~1100 amino acids (Fig 1A) and is thought to be key for Abl function [28]. Yet those few regions exhibiting significant sequence conservation only play minor roles in Abl function, and the role of the CTD remains poorly understood. Accordingly, we elected to undertake a systematic analysis of the entire C-terminal domain, without relying on any assumptions about the function of any particular stretch. Taking care to avoid disrupting previously identified motifs or domains (Fig 1B), we subdivided the CTD into smaller regions (quarters, or 1-4Q), and systematically deleted these regions of Abl, creating AblΔ1Q through AblΔ4Q. Conversely, we added these selected regions back to the N-terminal region of Abl (creating N-1Q through N-4Q), to determine which regions are by themselves sufficient to restore function to the kinase-active half of the molecule. Transgenes also include a wild type Abl, as well as constructs expressing only the N-terminal (AblN) or C-terminal (AblCTD) region. All transgenes were inserted via PhiC31-mediated transgenesis into the *ZH-attP-22A* landing site [46] to simplify genetic manipulation and minimize position effects on transgene expression (see S1 Fig for expression data). Having created this battery of Abl mutants, we then tested how manipulation of these CTD regions impacts the ability of our transgenes to rescue *Abl* axon guidance defects and adult lethality.

### The first quarter of the Abl C-terminus is critical for Abl function

**Fig 2:**
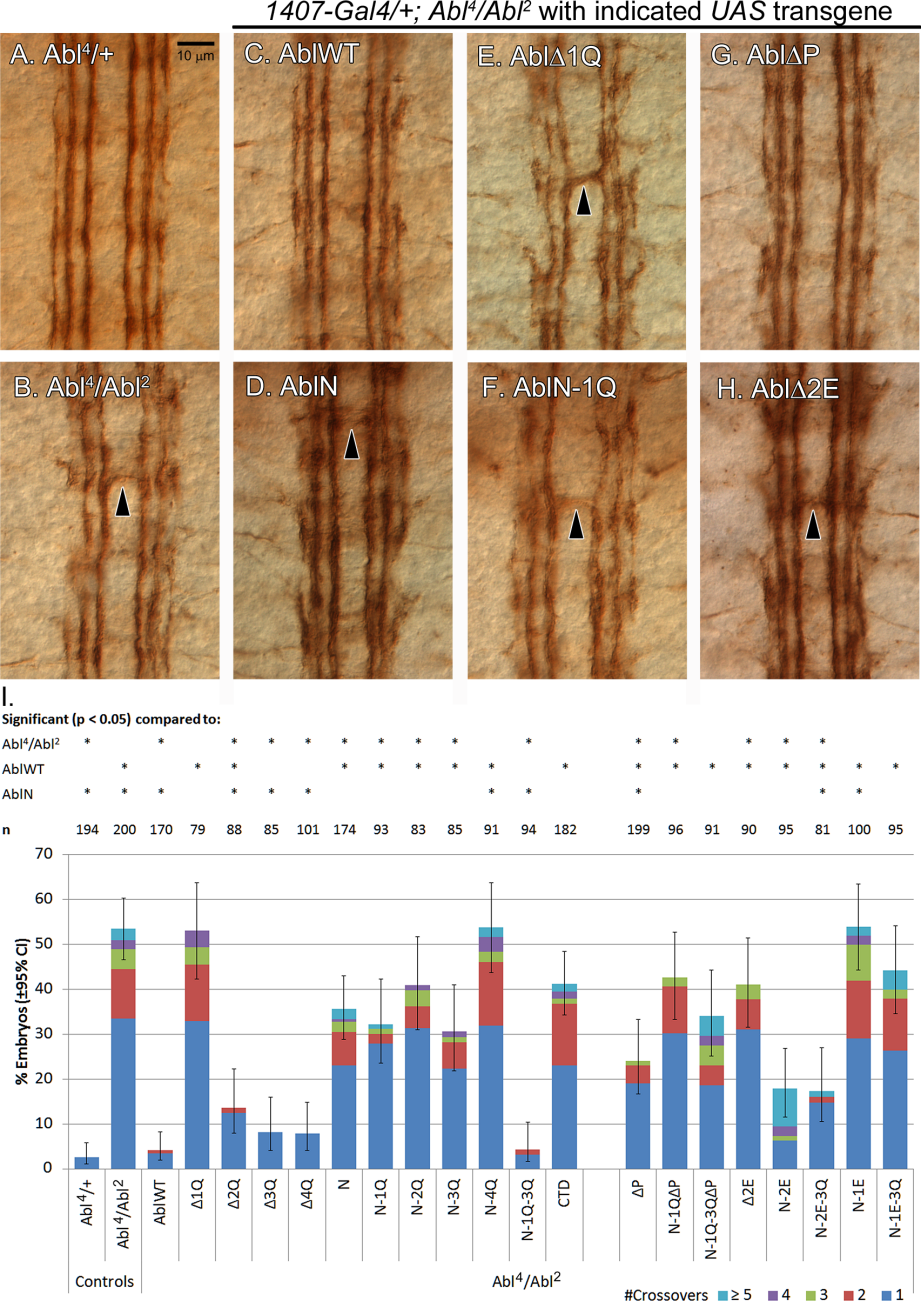
Rescue of midline axon guidance defects in homozygous *Abl* mutants with Abl transgenes. Mutant transgenes were expressed pan-neurally with the *1407-Gal4* driver, and nerve cords of stage 16–17 embryos were examined for crossing over defects after immunostaining for mAb 1D4. (A) *Abl* heterozygous embryo, which has a wild-type phenotype showing 3 longitudinal fascicles on each side of the midline. (B) *Abl*^*4*^*/Abl*^*2*^ embryo showing midline crossing over defects (arrowheads). (C-H) Rescue of *Abl*^*4*^*/Abl*^*2*^ defects by expressing of the indicated transgene: (C) *AblWT*, (D) *AblN*, (E) *AblΔ1Q*, (F) *AblN-1Q*, (G) *AblΔP*, and (H) *AblΔ2E*. The *AblWT* transgene (C) almost fully rescues crossing over defects. *AblΔ1Q* (E) completely fails to rescue defects while the other transgenes give varying degrees of partial rescue ranging from moderate (*AblΔP*) to low (*AblN*, *AblN-1Q*). (I) Quantification of crossing over defects. ≥79 embryos were scored over 3 replicates per genotype. Asterisks (*) over a particular column indicate significant difference (p < 0.05) compared to the indicated transgene or genotype. Deletion of 1Q totally abolishes rescue, while the *AblN-1Q-3Q* transgene is sufficient for rescue at a wild-type level. Mutants that have 1E, 2E or P removed partially impair rescue.

Zygotic loss of Abl causes ectopic midline crossing errors in the embryonic nerve cord, as quantified in late-stage embryos stained for the axonal marker Fasciclin 2 (mAB 1D4). This antibody labels 3 longitudinal fascicles on each side of the midline within the nerve cord, not crossing the midline when viewed in wild type stage 16/17 embryos (Fig 2). However, in *Abl* zygotic mutants, roughly 50% of embryos show one or more ectopic midline crossovers that are Fas2-positive, a phenotype that serves as the basis for our *Abl* rescue assay. As expected, we can rescue these defects almost fully by pan-neural expression of our wild-type Abl transgene using the *1407-Gal4* driver. When *Abl* transgenes from our CTD deletion series were expressed, transgenes carrying deletions of 3Q or 4Q rescue midline crossover defects to a degree indistinguishable from the wild-type transgene, while deletion of 2Q marginally impairs rescue (~15% crossovers). Remarkably, the AblΔ1Q transgene fails entirely in rescuing these defects (Fig 2C and 2I).

On the other hand, while pan-neural expression of AblN partially rescues some of the ectopic crossovers observed in *Abl* mutant embryos (~35% embryos with defects), the addition of 1Q, 2Q or 3Q does not significantly improve rescue by AblN, while adding 4Q to AblN abolishes the small amount of rescue seen with AblN alone. Strikingly, rescue is fully restored if both 1Q and 3Q are added to Abl-N, indicating that the 1Q and 3Q regions together provide the majority of the Abl CTD’s function in axon guidance at the midline. Together, these data point to a key role for 1Q in axon guidance, although this region alone is not sufficient to restore function to AblN unless 3Q is included.

### Rescue of Abl adult lethality

The relative robustness of Abl to removal of individual CTD quarters in the rescue of *Abl* axon guidance defects was surprising. Thinking that the maternal contribution of Abl [5, 47] may have partially obscured our ability to uncover roles for these regions, we decided to next assess the ability of our mutants to rescue adult lethality. Pupal development is known to represent a significant lethal phase for *Abl* mutants, with only a small number of escapers surviving this step in development [48]. Examination of lethality at this stage likely minimizes the effect of the maternal contribution of Abl.

Endogenous Abl is widely expressed at low levels in most tissues [49], suggesting that a non-neuronal role for Abl might contribute to adult lethality. However, ubiquitous expression with *Act5C-Gal4* produces very few surviving adults (S2A Fig), although some rescue (~15–30%) was provided by *AblΔ4Q*, *AblN-1Q*, and *AblN-1Q-3Q*, all of which lack 4Q while retaining 1Q. As 4Q includes the FABD, these data seem to suggest that the toxic effect of ubiquitously over-expressed Abl might be associated with its ability to interact with actin. Given the propensity to cause lethality for most transgenes, we did not pursue rescue with this *Act5C-Gal4* driver line further.

**Fig 3:**
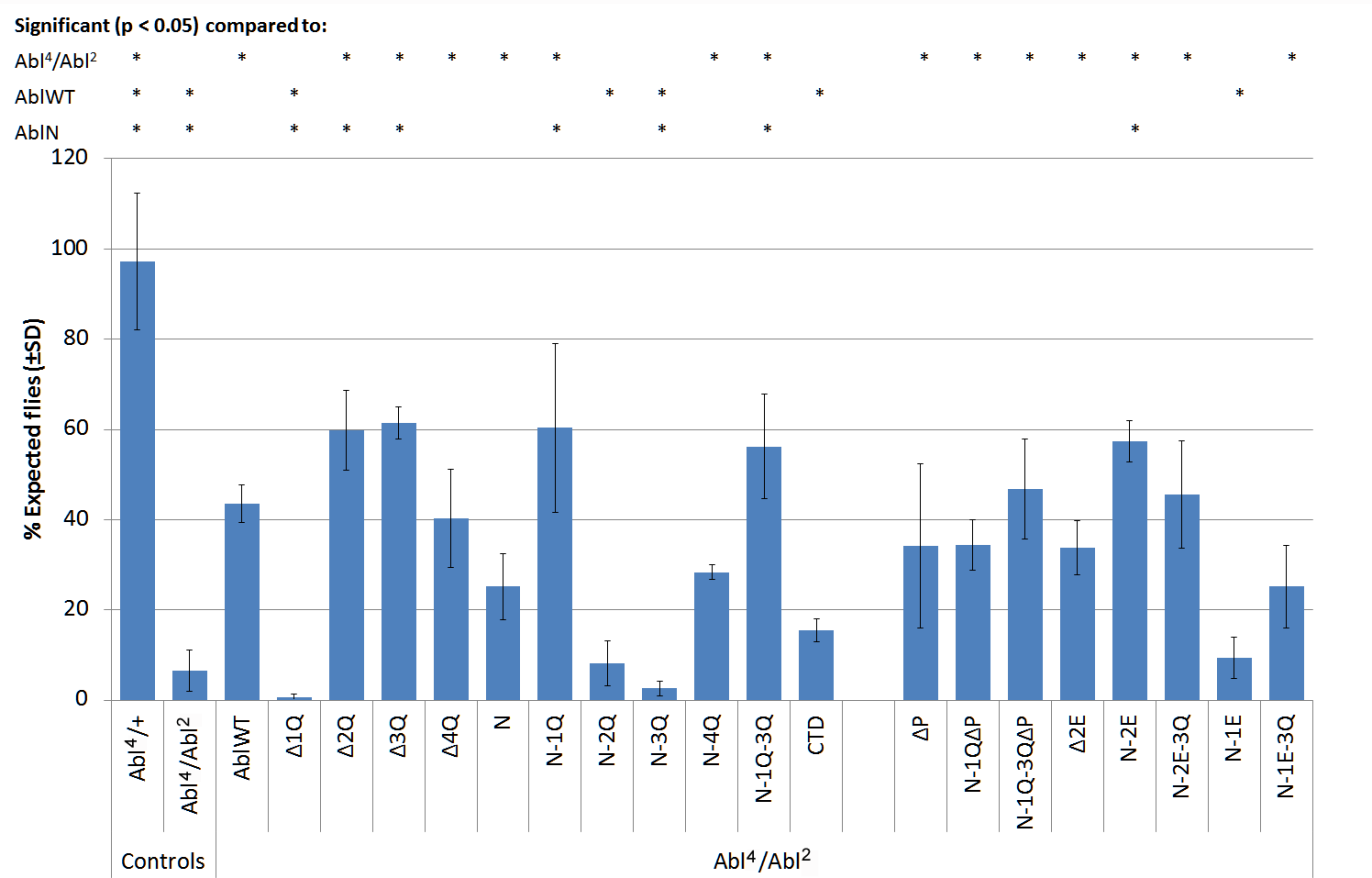
Rescue of adult lethality in homozygous *Abl* mutants by Abl transgenes expressed with the *1407-Gal4* driver. Asterisks (*) over a particular column indicate significant difference (p < 0.05) compared to the indicated transgene or genotype. Three replicates of ≥400 flies were counted, with approximately 50 flies for each of males and females in the non-expressing *Abl* heterozygous category. This category was used to normalize rescued *Abl* mutant flies. Deletion of 1Q totally abolishes rescue, while addition of 1Q to AblN gives rescue at the level of the wild-type transgene. Mutants that have 1E, 2E or P removed have small effects on rescue.

Instead, we focused on the importance of neuronal function of Abl in *Abl* adult lethality. Pan-neuronal expression of wild-type *Abl* with *1407-Gal4* rescues about half of the observed lethality in *Abl* mutant flies. Consistent with rescue of axon guidance defects, only removal of 1Q abolishes rescue (Fig 3A), and only the addition of 1Q to the N-terminus restores wild-type viability to AblN.; yet, AblN-1Q is not substantially better than AblN at rescuing axon guidance defects. Similar to rescue of axon guidance defects, AblN-1Q-3Q remains a minimally effective Abl restoring adult viability to the same level as observed for wild type Abl.

It is worth noting that pan-neural expression of the *AblΔ1Q* transgene actually induces lethality in *Abl* heterozygotes (over 60%, S2C Fig). We suspect that this lethality occurs post-embryonic development since the nerve cords of wild type or *Abl* heterozygous embryos still look normal when *AblΔ1Q* is expressed in all neurons. Heterozygous lethality is the first evidence of a gain-of-function effect for the *AblΔ1Q* transgene, suggesting that the transgene may be aberrantly regulated in at least one critical Abl function, or be incorrectly expressed in a subset of neurons.

To address this latter issue, we attempted to better replicate parts of the endogenous Abl expression pattern by using two other *Gal4* lines controlled by *Abl* enhancer fragments, *R60H11* and *R65D01* [50]. Both of these *Abl-Gal4* enhancers drive neuronal expression, although in a smaller subset of neurons and at lower levels than observed with *1407-Gal4* (S3 Fig). For unknown reasons, rescue with these drivers are biased towards adult males, and thus only males are used here for comparison. Considering only males, the rescue observed with both these driver lines is similar in pattern to that observed using the *1407-Gal4* driver, with a couple of notable exceptions (S2B Fig). Most interestingly, the *AblΔ1Q-*dependent lethality observed with *1407-Gal4* is lost using either of the Abl driver lines, suggesting that some cell-specificity or expression level control is important. In addition, *AblN* alone failed to rescue with both Abl drivers even though it partially rescues (30%) with *1407-Gal4*, and a few subtle differences occur as different quarters are added to AblN. A dominant phenotype also emerged using the *60H11* driver, as *Abl* heterozygous flies expressing *AblN*, *AblN-2Q* or *AblN-3Q* fail to carry out wing expansion. This has not been pursued further.

Taken together, it is clear that 1Q plays the largest role in rescue of adult lethality, consistent with rescue of *Abl* axon guidance defects. However, Abl-dependent viability is not always associated with its role in axon guidance (e.g. AblN-1Q), and the removal of 2Q and 4Q regions, which are relatively benign during axon guidance, can subtly alter viability, suggesting that these regions are not simply inert.

### Distinct regions of the 1^st^ quarter contribute towards Abl function

The observations that *AblΔ1Q* completely fails to rescue both *Abl* axon guidance defects and adult lethality, while addition of 1Q or 1Q-3Q to AblN results in functional *Abl* transgenes points towards a critical role of the 1Q region for Abl function. By comparative sequence analysis with Abl proteins of other *Drosophila* species, the 1Q region of the Abl CTD can be roughly subdivided into two halves of low and high conservation (1^st^ and 2^nd^ eighths, or 1E and 2E), respectively (Fig 1C). Additionally, the highly conserved half (2E) contains a single canonical PxxP motif (PAPPKR, a.a. 769–774 in Uniprot accession number# M9PFS1) that aligns to a PxxP motif in the CTD of vertebrate Abl homologs. An *Abl* transgene lacking a region roughly equivalent to our 2E region was previously demonstrated to be highly impaired in rescuing Abl-dependent morphogenetic processes [6]. In that work, the PxxP motif was suggested, but not tested, to be the key feature of this region, as PxxP motifs are the minimal consensus motif postulated to be required for interaction with SH3 domains found in certain Abl partners [39, 51, 52].

To specifically test the role of PxxP motif, we created a set of Abl transgenes deleting the PxxP motif (ΔP) in either full length *Abl*, *AblN-1Q* or *Abl-1Q-3Q*. Our rationale for introducing these deletions into *AblN-1Q* and *Abl-1Q-3Q* is based on the observation that the Abl CTD contains 3 other PxxP sequences that fit the canonical type I or type II SH3-binding consensus motifs [38, 39]; these additional motifs are located in the 2^nd^ and 4^th^ quarters of the CTD (Fig 1A). Recall that the *AblN-1Q* and *AblN-1Q-3Q* transgenes substantially rescued *Abl* loss in our prior *Abl* rescue assays. Therefore, the removal of the 1Q PxxP motif in these minimal transgenes should uncover roles for this particular motif while avoiding any possible redundant contributions from the other PxxP motifs. Along the same lines, we also examined the role of the 1E and 2E regions by deleting them in the same set of transgenes, producing *AblΔ2E*, *AblN-1E*, *AblN-2E*, *AblN-1E-3Q* and *AblN-2E-3Q*.

In terms of axon guidance defects, removal of the PxxP motif reduces, by about half, the ability of full-length Abl to rescue these crossover defects, and reduces the rescue of *AblN-1Q-3Q* from near wild-type to the level of *AblN* alone (Fig 2I). This effect is similar but stronger with all transgenes lacking 2E (which contains the PxxP motif), with *AblΔ2E*, *AblN-1E-3Q* and *AblN-1E* rescuing only at the level of *AblN* alone, or worse. Conversely, *AblN-2E-3Q* partially rescued defects, although not at the level of either *AblN-1Q-3Q* or wild-type *Abl*. Clearly the PxxP motif is important for Abl function during axon guidance, but the surrounding 2E sequence provides additional function. On the other hand, the severity of the Δ1Q mutation cannot be explained either by the loss of the PxxP motif alone or by loss of 2E, and indicates that 2E and its PxxP motif must still cooperate with 1E to provide full wild-type function.

Interestingly, in terms of adult lethality, pan-neural expression of all mutant transgenes, except AblN-1E rescues adult lethality indistinguishable from the wild-type transgene (Fig 3); AblN-1E only allows a few adults to survive. Curiously, while AblN-2E-3Q rescued *Abl* adults to the same level as wild-type Abl, it was uniquely the only transgene of this series to cause substantial adult lethality in *Abl* heterozygotes (35% lethality, S2C Fig); this parallels the induced lethality observed with *AblΔ1Q*. Adult rescue using the *1407-Gal4* driver is largely consistent with rescue using the *60H11* and *65D01* Abl drivers (S2B Fig), although some minor differences suggest increased sensitivity to the driver’s exact expression pattern (or level). We also note that flies expressing *AblN-2E* and *AblN-2E-3Q* with the *60H11* driver fail to expand their wings. This is despite the *AblN-2E* and *AblN-2E-3Q* transgenes rescuing adult lethality at levels close to that observed for the wild-type transgene, showing that they too are slightly impaired in function when 1E is removed (S2B Fig).

In summary, the 2E region and its PxxP motif are the most important region of 1Q, as any transgene that have these motifs removed are impaired in axon guidance. Moreover, transgenes that have 2E deleted (*AblΔ2E*, *AblN-1E* and *AblN-1E-3Q*) do not rescue axon guidance defects as well as their counterparts where only the PxxP motif was removed (*AblΔP*, *AblN-1QΔP* and *AblN-1Q-3QΔP*). These data support our contention that the context of the PxxP motif (i.e. 2E) is important for function, which is consistent with Rogers et al’s [6] report on the importance of the equivalent CR1 region. Moreover, as adult rescue appears to be more resistant to removal of these regions, 2E and the PxxP motif may participate only in some of the differential roles of Abl, something that future work will have to explore in more detail. Finally, there is some indication that both AblΔ1Q and AblN-2E-3Q are functional proteins that have gain-of-function effects resulting from the removal of 1E.

### Gain-of-function assays reveal further roles for the CTD region

Abl is known to function downstream of numerous guidance receptors during formation of the nerve cord [12–17]. Thus, it was surprising that the 2Q, 3Q and 4Q regions, which together span most of the CTD, are individually dispensable during rescue of *Abl* axon guidance. To further assess the role of the CTD in axon guidance, we turned to an over-expression assay in sensitized genetic backgrounds. Overexpression of Abl in a wild-type background only yields a few subtle phenotypes in a small subset of motoneurons [53], yet when over-expressed in *frazzled* (*fra*) homozygous [15] or *slit* heterozygous [14] mutants, leads to gross exacerbation of nerve cord defects. These are dominant phenotypes that occur even with a full complement of maternal and zygotic Abl. Admittedly, we do not yet know much about the molecular mechanisms underlying these defects, but nevertheless, we sought to use the increased sensitivity to Abl signaling to reveal additional contributions of the CTD to Abl function. Indeed, these assays proved to be helpful in identifying contributions of all four quarters during axon guidance.

**Fig 4:**
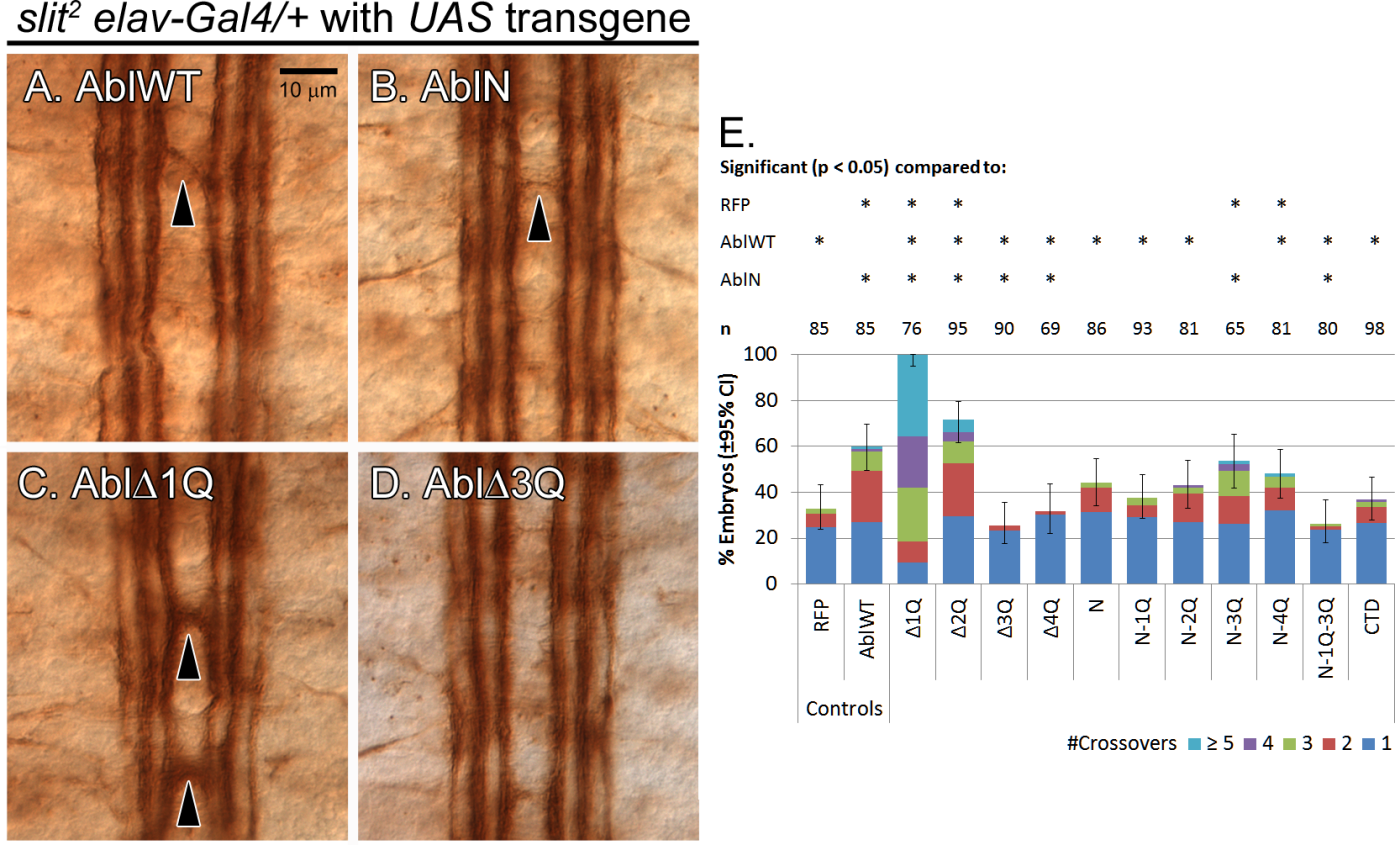
Gain-of-function assay for Abl transgenes in a *slit*^*2*^ heterozygous background. Abl mutant transgenes were overexpressed pan-neurally in heterozygous *slit*^*2*^ embryos with the *elav-Gal4* driver, and nerve cords of stage 16–17 embryos were examined for crossing over defects via immunostaining for mAb 1D4. (A-D) Overexpression of transgenes causes midline crossing overs (arrowheads) in a *slit*^*2*^ heterozygote. Expression of *AblWT* (A) and *AblN* (B) causes moderate crossing over defects. Deletion of 1Q in Abl (C) causes severe crossing over defects, while deletion of 3Q (D) abolishes Abl-induced crossing over defects. (E) Quantification of crossing over defects based on ≥65 embryos per genotype. Asterisks (*) over a particular column indicate significant difference (p < 0.05) compared to the indicated transgene or genotype. *AblΔ1Q* uniquely causes defects in 100% of embryos examined. Of the other transgenes, only *AblΔ2Q*, *AblN-3Q* and *AblN-4Q* cause significantly higher defects than the *RFP* negative control.

The repulsive receptor Robo and its ligand Slit are major players in repulsive axon guidance at the *Drosophila* midline, and recruits Abl as one of its downstream effectors [54]. A heterozygous loss of the midline repellent *slit* cause ectopic midline crossovers in the embryonic nerve cord, and the frequency of these increases with over-expression of Abl transgenes [14]. Thus, over-expression of Abl is thought to alter midline repulsion signaling. Here we assessed how our Abl transgenes alter midline guidance by overexpressing them in all neurons of *slit* heterozygous embryos using the *elav-Gal4* driver [14] and scoring ectopic crossovers using mAb 1D4 (Fig 4).

First, we established a baseline level of midline axon guidance defects for the *slit* heterozygous embryos by overexpressing an RFP transgene inserted at the 22A site. About 30% of all *slit* heterozygous embryos exhibit 1 or 2 ectopic crossovers when the RFP control is expressed, and overexpression of wild type Abl doubles these crossovers. Removal of 1Q from Abl severely enhances the midline crossover phenotype (to 100% penetrance), while removal of 3Q instead suppresses this phenotype back to the baseline level. Interestingly, removal of 4Q also suppresses these Abl-dependent ectopic crossovers, while removal of 2Q mildly enhances the frequency of crossovers over wild-type Abl. The 2Q and 4Q phenotypes are unique to the *slit* assay, and may reflect the selective requirement of these regions during midline repulsive signaling. Of the AblN series, only the addition of 3Q to AblN significantly increases ectopic crossovers to roughly the levels observed with wild-type Abl. On the other hand, even though AblN-1Q-3Q mutant was minimally sufficient to rescue Abl axon guidance defects, it is not capable of inducing ectopic crossovers in the *slit* heterozygote. This points to an interesting interaction between the first and third quarters.

Next we turned to midline attraction using a *frazzled* mutant background. The attractive Netrin receptor Frazzled recruits Abl as a downstream effector [13, 27], although the precise role of Abl in this pathway is not well understood. When wild-type Abl is overexpressed in *fra* homozygous embryos, axon tracts of the nerve cord exhibit many defects, including a loss in posterior commissure formation and an increase in the number of gaps in the longitudinal connectives [15]. In previous work, we had postulated that the development of the ventral cord becomes highly sensitive to Abl signaling levels in the absence of Fra, resulting in numerous defects as Abl presumably began to function incorrectly downstream of other unknown receptors [15]. As such, we anticipated that a *fra* background might be ideal for picking out subtle differences in the function of our Abl transgenes.

**Fig 5:**
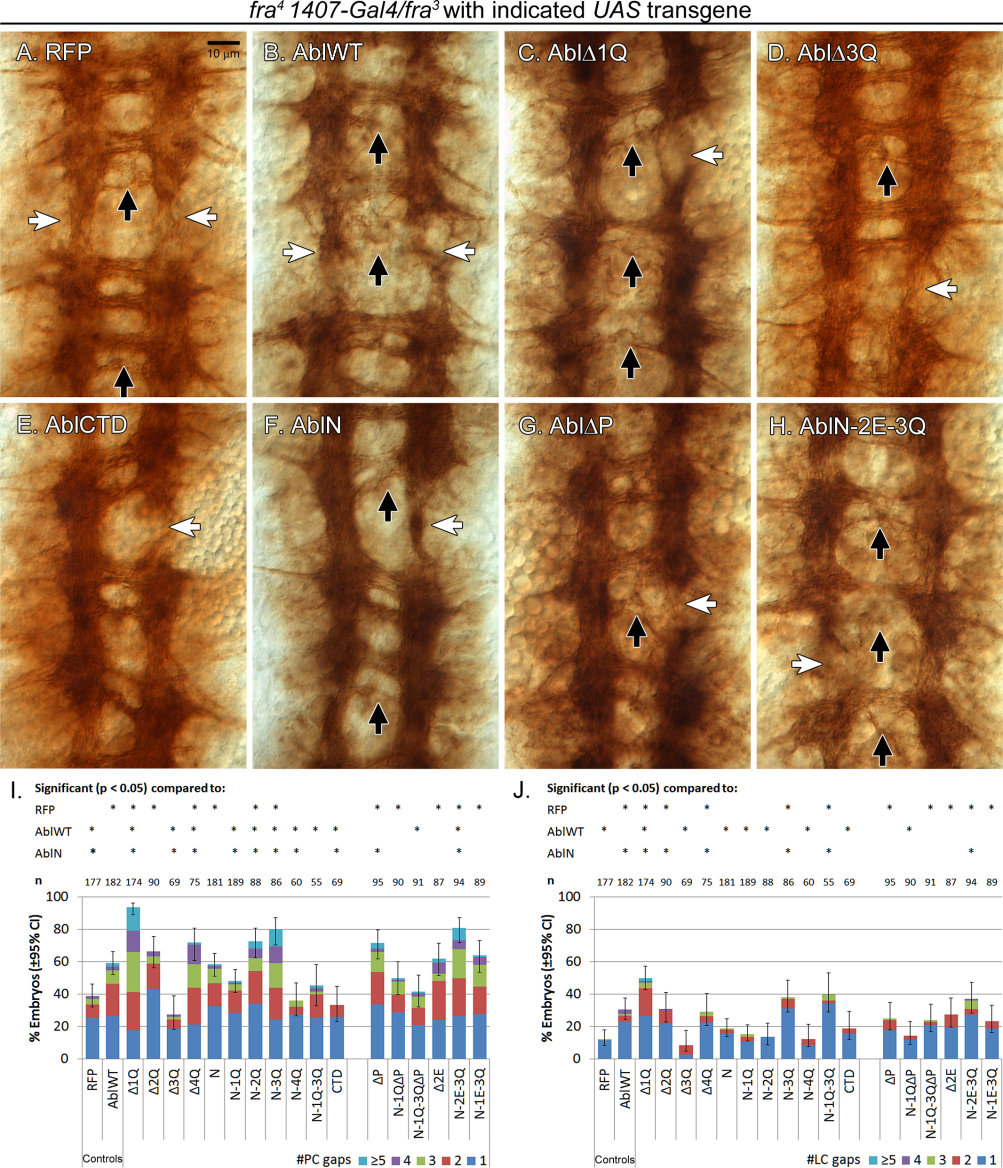
Gain-of-function assay for Abl transgenes in a homozygous *fra* mutant background. Mutant Abl transgenes were overexpressed pan-neurally in *fra* homozygous embryos with the *1407-Gal4* driver, and nerve cords of stage 15–17 embryos were examined for crossing over defects after immunostaining for mAb BP102. (A-H) Exacerbation of *fra* nerve cord defects by expression of Abl transgenes. (A) Control *fra* phenotype with expression of *RFP* showing baseline level of thinning/gaps in posterior commissures (black arrows) and thinning/gaps in longitudinal connectives (white arrows). *AblWT* (B) expression causes an increase in defects in posterior commissures and longitudinal connectives, and this is mirrored by *AblN* (F) and *AblΔP* (G), while *AblΔ3Q* (D) and *AblCTD* (E) have no effect on the *fra* phenotype. Both *AblΔ1Q* (C) and *AblN-2E-3Q* (H) cause an increase in defects more severe than *AblWT*. (I) Quantification of posterior commissure (PC) gaps (I) and longitudinal connectives (LC) gaps (J) in *fra* mutants overexpressing Abl transgenes based on ≥55 embryos scored per genotype.

We overexpressed our series of transgenes in *fra* homozygotes with *1407-Gal4*, and quantified axon guidance defects in stage 16–17 embryos immunostained with the mAb BP102, which highlights the ladder-like structure of the axon tracts of the embryonic nerve cord (Fig 5). First, we established a baseline for the *fra* homozygous embryos by overexpressing an RFP transgene inserted at the 22A site. At this baseline state, about 40% of embryos exhibit a loss or thinning of posterior commissures, and this almost doubles when the wild-type *Abl* transgene is overexpressed. A similar trend is seen when gaps in the longitudinal connective are scored. While other guidance defects were also scored (see S1 Table), we focused on the formation of the commissures and longitudinal connectives as Abl has been linked to their development [5, 15, 23, 27].

Unlike wild-type *Abl*, *AblCTD* alone did not enhance axon guidance defects in *fra* mutants, while *AblN* had differential effects on commissures and longitudinal connectives. Removal of 2Q had no effect while removal of 4Q slightly increased the number of posterior commissure gaps. Strikingly, deletion of 1Q significantly enhances (to 95%) the severity of posterior commissure gaps, and increases longitudinal defects to 50%, while deletion of 3Q prevents Abl from inducing these defects. Addition of either 1Q or 4Q instead suppresses the ability of AblN to induce defects in *fra* mutants, while differential effects on commissures and longitudinal connectives are seen with the AblN-2Q and AblN-1Q-3Q transgenes. Thus, by using the *fra* background, we uncover evidence indicating that each quarter contributes functionally to Abl as it presumably cooperates with various guidance receptors during formation of commissures or longitudinal connectives.

Given the severity of phenotypes observed when *AblΔ1Q* is overexpressed, we also assessed our series of 1Q mutants in the *fra* background. Surprisingly, deletion of the PxxP motif in wild-type *Abl*, *AblN-1Q* or *AblN-1Q-3Q* had no effect on the ability of our transgenes to induce defects. Similarly, neither *AblΔ2E* nor *AblN-1E-3Q* was substantially different from their counterparts wild-type *Abl* and *AblN-1Q-3Q*, respectively. Despite the fact that the comparable *AblN-1Q-3Q* is less effective than wild-type *Abl* at inducing defects, the *AblN-2E-3Q* transgene caused defects at a higher level (80%) than wild-type *Abl*, even though it is still capable of partially rescuing both adult lethality and axon guidance defects in our earlier assays. These paradoxical results await further investigation, but do suggest that AblN-2E-3Q may be partially misregulated similar to but not to the same extent as AblΔ1Q.

Together the severity of defects in these fra mutants reinforce the importance of 1Q in Abl function, and support the premise that this *Abl* transgene is inappropriately regulated. This dysregulation does not appear to be caused by deletion of either the PxxP motif or the 2E region, indicating an involvement of the entire 1Q region in Abl function. Moreover, it is clear that deletion of other quarters also show differential effects on Abl function, especially that of 3Q which suppresses Abl’s ability to cause phenotypes in both *slit* and *fra* backgrounds. As described below, this region appears to be important for localization of Abl to axons.

### The 3^rd^ quarter of the CTD localizes Abl to axons

**Fig 6:**
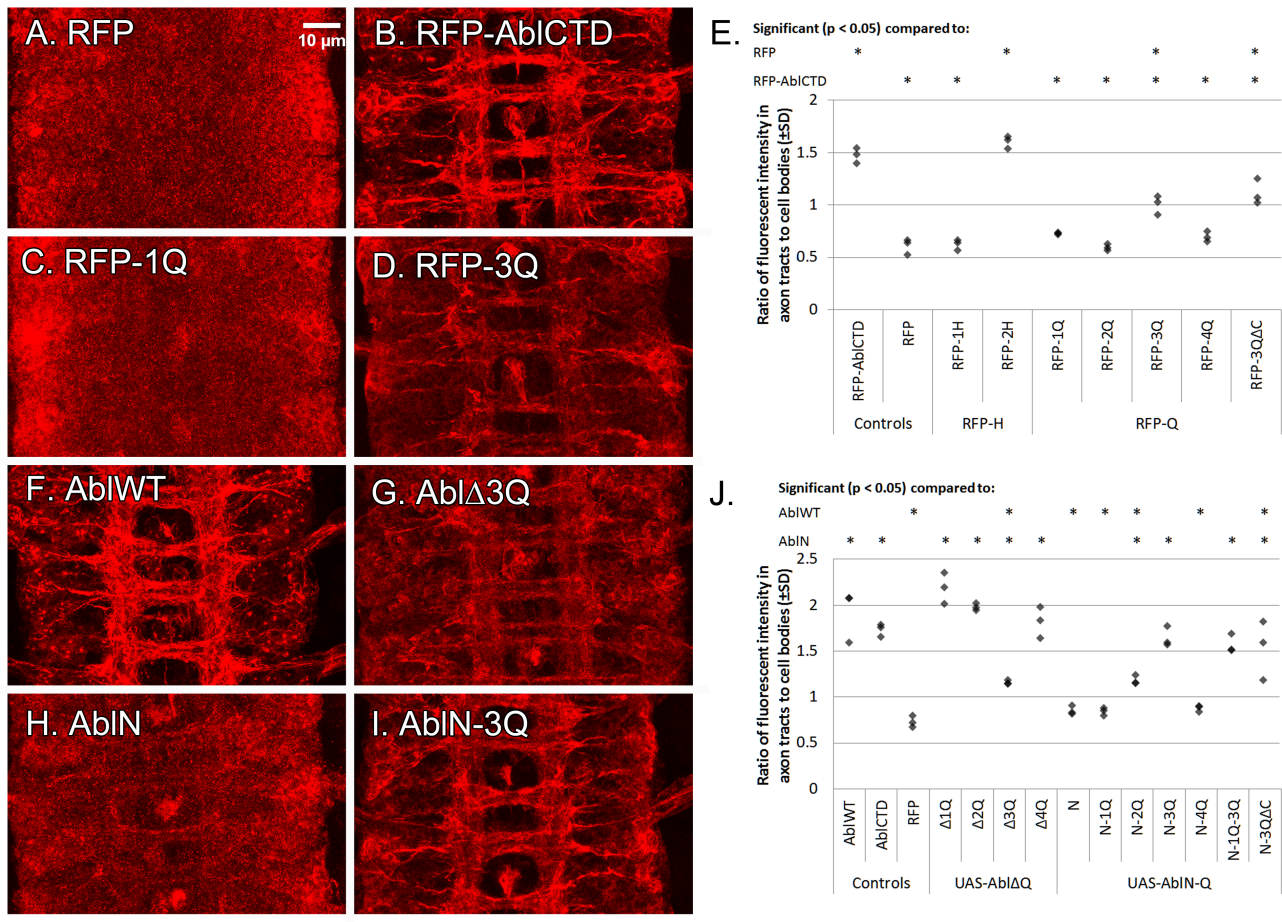
Axonal localization of Abl transgenes. Transgenes were expressed pan-neurally with *1407-Gal4*, and immunostained for the C-terminal FLAG tag. The RFP control (A) is present mainly in cell bodies, while RFP fused to AblCTD (B) localizes strongly to axons. RFP fused to 1Q (C) fails to localize to axons, as do 2Q and 4Q (not shown), but RFP fused to 3Q (D) is localized to axons. (E) Quantification of localization. Areas of axon tracts and cell bodies were defined in flattened confocal stacks for 3 different embryos, and the ratio of fluorescent intensity in axon tracts vs cell bodies was taken as described in Methods. Asterisks (*) over a particular column indicate significant difference (p < 0.05) compared to the indicated transgene or genotype. RFP-AblCTD and RFP-2H localize to axons strongly. RFP-3Q localizes to axons less strongly, and removal of a region containing the EVH1-binding motifs (ΔC) does not further change its localization. Full-length Abl (F) localizes strongly to axons, and deletion of 3Q in an Abl transgene (G) reduces this localization. AblN alone (H) localizes weakly to axons, but addition of 3Q to AblN (I) significantly restores axonal localization. (J) Quantification of localization, with statistics as in (E). Removal of 3Q, but not 1Q, 2Q or 4Q, from full-length Abl significantly reduces axonal localization. Addition of 3Q in AblN-3Q and AblN-1Q-3Q is sufficient to restore near-wildtype localization. Removal of the EVH motifs (ΔC) does not change localization of AblN-3Q.

During our initial experiments, we assessed the level of Abl transgene expression by immunostaining using the FLAG epitope present at the C-terminus of our transgenes. Wild-type Abl localizes strongly to the axon tracts of the nerve cord, confirming prior reports [27, 28, 49], and localization remained strong even with removal of the EVH1-binding motifs and FABD. Furthermore, while AblN alone localizes poorly to axons, AblCTD is sufficient on its own for strong axonal localization indistinguishable from wild-type Abl (see Fig 6). In support of this, protein produced from the *Abl*^*1*^ allele, which introduces a stop codon after the kinase domain, and an Abl transgene substituting the murine Abl CTD for the *Drosophila* CTD, also fail to localize to axons [28]. These data suggest that localization of Abl to axons is a major function of the *Drosophila* Abl CTD, and we set out to define the regions of the CTD responsible for localizing Abl to axons.

First, we generated and expressed a series of transgenes fusing RFP to FLAG-tagged fragments of the Abl CTD and assessed which region(s) was sufficient to localize RFP to axons. The CTD was divided into approximate halves (1-2H) and then quarters (1-4Q, also see Fig 1B). The RFP transgenes were then inserted via PhiC31-mediated transgenesis into the *ZH-attP-86Fb* landing site [46], and expressed in the embryo with *1407-Gal4*. As the intrinsic fluorescence of our RFP transgenic proteins was low, to quantitatively assess the degree of localization of transgenic protein, we carried out fluorescent immunohistochemistry on embryos using anti-FLAG and mAb BP102 antibodies, and took confocal image stacks for at least 3 stage 16–17 embryos per transgene. For each embryo, the area in the flattened confocal stack corresponding to axons in the CNS was defined by the presence of BP102 staining, and the mean intensities within axons and cell bodies determined. The ratio of mean intensity within axons to mean intensity in cell bodies was then taken as a measure of localization.

When expressed in the embryonic nerve cord, only RFP transgenes fused to full-length AblCTD, 2H or 3Q were capable of localizing to axons (Fig 6A–6E). RFP alone localized poorly to axons (axon to cell body ratio of ~0.6). Interestingly, quantification of localization showed that while localization of RFP-2H was indistinguishable from the full-length RFP-AblCTD (ratio of ~1.5), RFP-3Q localized to axons about half as well (ratio of ~1). Thus, both quarters (3Q and 4Q) found in RFP-2H cooperate in axonal localization, although RFP-4Q alone is insufficient. Within the 3Q region, the conserved EVH1-binding motifs are the only recognizable motifs of significant interest. However, localization of 3Q remains intact even if the EVH1-binding motifs are removed (ΔC, also see Fig 1D). Thus, the 3^rd^ quarter, but not the EVH1-binding motifs, is sufficient to direct a heterologous protein to axons.

Next, we sought to test the significance of each quarter in the context of full-length Abl using our set of mutant Abl transgenes (Fig 6F–6J). In embryos, both full-length Abl and AblCTD were enriched in the axon tracts of the nerve cord as expected (ratio of ~1.8), while very little AblN is localized to axons (ratio of 0.8). Deletion of 1Q, 2Q or 4Q had no noticeable impact on localization of the protein. On the other hand, deletion of 3Q significantly impaired axonal localization (ratio of 1.15) compared to the wild-type Abl protein, but did not fully abolish it as compared to AblN alone (ratio of 0.8). Furthermore, addition of 3Q to AblN, but not any other quarter, resulted in significant restoration of axonal localization (ratio of 1.65). This localization remains intact even if the EVH1-binding motifs are removed from the 3Q region (ΔC).

Thus, the 3Q region of the Abl CTD is sufficient for localization by itself to axon tracts, and is partially required for localization in the context of full-length Abl. The ability to recruit Abl to axons where it is required for axon guidance is consistent with the ability of the AblN-1Q-3Q transgene to rescue *Abl* guidance defects despite the inability of AblN-1Q to do so. Impaired localization may also explain the observation that AblΔ3Q induces fewer axon guidance phenotypes in the *slit* and *fra* backgrounds, as well as the increased ability of AblN-3Q to cause defects in these genetic backgrounds.

### The 1^st^ and 3^rd^ quarters of the AblCTD are disordered

**Fig 7:**
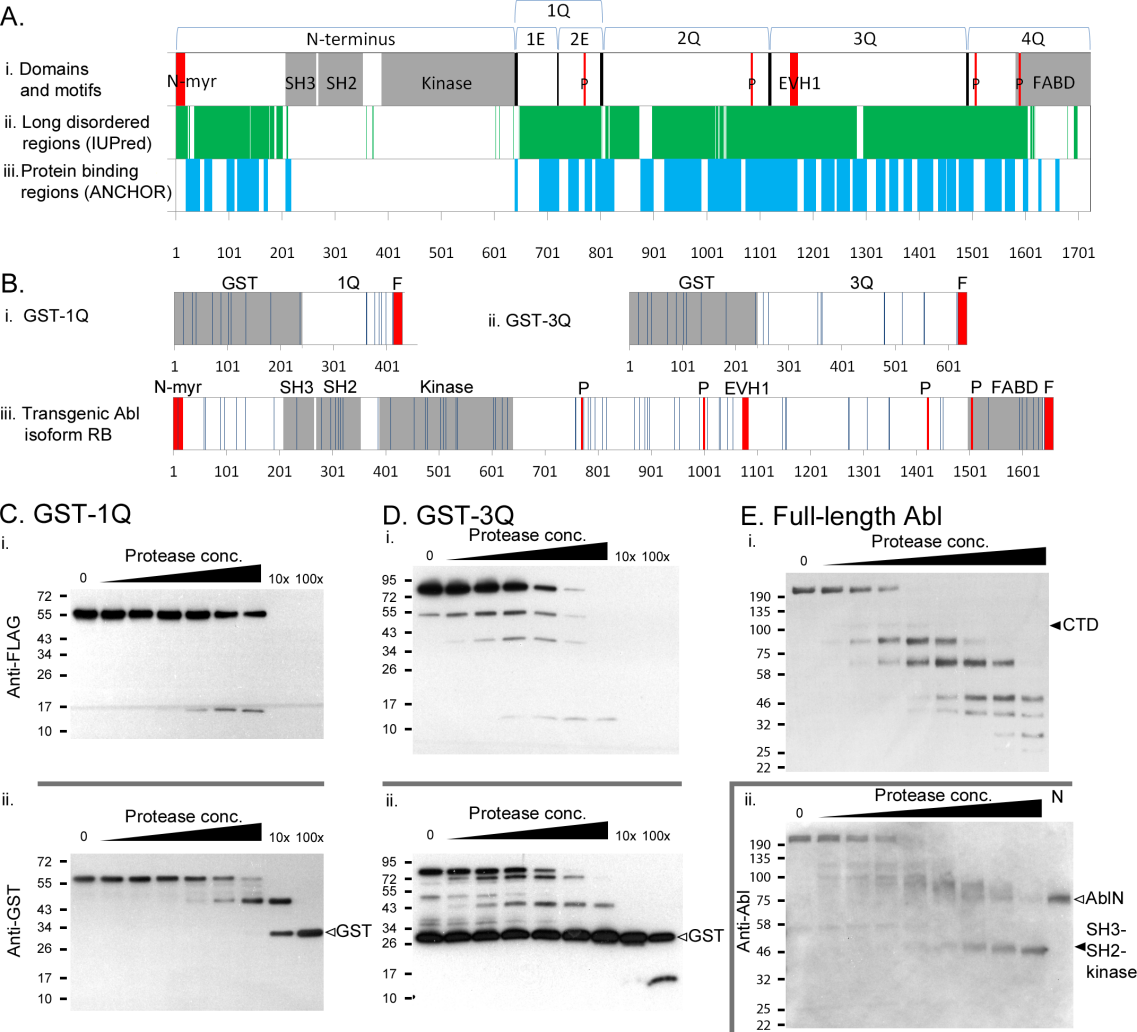
Protease susceptibility assay reveals disorder in the Abl CTD. (A) Predicted intrinsic disorder (IUPred) and protein binding regions (ANCHOR) within Abl. (B) Predicted clostripain (endoproteinase Arg-C) digestion sites within GST-1Q, GST-3Q and full-length Abl for protease susceptibility assay. Sites are well distributed over both the globular domains and predicted disordered regions, and sites within disordered regions should be preferentially utilized. Clostripain digest of purified GST-1Q (C) and GST-3Q (D) with 6 lanes of increasing doses, and two final lanes with 10x and 100x protease as indicated. All FLAG-tagged fragments are more than ~25kD smaller than the full-length protein, and no GST-tagged fragments are smaller than the ~25kD GST domain (▻) except at the 100x protease concentration for GST-3Q. Both indicate that protease sites within the CTD fragments are preferentially utilized by clostripain, compared to the GST domain. (E) Clostripain digest of full-length transgenic Abl in total cell lysates of 3^rd^ instar CNS. FLAG-tagged fragments are present at the size of the full-length CTD (~108 kD) or smaller, consistent with preferential digestion within the CTD. Fragments labeled with a polyclonal antibody against the Abl N-terminus show either fragments at or above the size of full-length AblN (~69 kD, ▻) or at the size of the SH3-SH2-domain (~49 kD, ►). In the last lane, AblN (N) alone was also expressed in 3^rd^ instar CNS, but the lysate was not subject to digestion.

The Abl CTD, including the 1Q and 3Q regions, is largely devoid of known or putative protein domains, except for the C-terminal FABD (Fig 1A). As such, it is not immediately obvious how 1Q and 3Q carry out their roles in Abl function and localization. To gain some insight, we undertook an extensive bioinformatic analysis of the CTD sequence using available online tools including SMART [55], CDD[56], JPred [57], and ELM [58]. Of these, the IUPred and ANCHOR prediction tools [59–62] suggested that the majority of the CTD, including 1Q and 3Q, is not only disordered, but may become ordered upon binding to other proteins (Fig 7A), thereby providing a mechanism where these regions could participate in protein-protein interactions. Accordingly, we set out to verify that these regions are indeed disordered using a protease sensitivity assay [63]. The principle of this assay is based on the observation that disordered regions are highly flexible and solvent-exposed, and are thus far more sensitive to proteolytic cleavage compared to globular regions. This selective susceptibility is easily demonstrated through partial proteolysis of the protein with carefully-titrated concentrations of protease.

To obtain a substrate for digestion, we fused the 1Q and 3Q regions to an N-terminal GST tag and retained a FLAG-tag on the C-terminus. For technical reasons, neither the full-length Abl protein nor the full-length Abl CTD could be bacterially expressed. Recombinant protein was expressed in *E*. *coli* and purified on glutathione agarose. Importantly, the globular GST domain of these recombinant proteins serves as an internal control in our protease sensitivity assay, as it is expected to be more resistant to cleavage compared to the putative disordered regions. The protease clostripain (Endoproteinase Arg-C), was chosen for this assay, as a similar number of cleavage sites are present in both GST and the 1Q and 3Q regions (10 in GST, 8 in 1Q, and 12 in 3Q; Fig 7B). Using both GST-1Q and -3Q, we titrated a range of protease concentrations that yielded a range of partial digest fragments.

The susceptibility of the 1Q and 3Q regions to digestion is readily observed using anti-FLAG and anti-GST western blots. The anti-FLAG immunoblots (Fig 7C and 7D) indicate that digestion at lower concentrations produce only a single digested FLAG-tagged fragment for GST-1Q, and a series of fragments for GST-3Q. The sizes of these digested fragments are consistent with the utilization of cut sites within the 1Q and 3Q regions, and not within the GST domain. Conversely, anti-GST immunoblotting reveals a series of GST-tagged fragments for both digests with sizes ranging between that of the full-length GST-1Q or 3Q, and GST alone, again consistent with the utilization of cut sites only within the CTD fragments. To further demonstrate the resistance of the GST domain relative to 1Q and 3Q, we carried out a more complete digestion with high protease concentrations (10x and 100x, as indicated on Fig 7C and 7D). At these concentrations, FLAG-tagged fragments are quickly lost, while a GST-tagged band remains at the expected size for the entire GST domain alone (28 kD) that only begins to show slight digestion at the highest protease concentration. Total protein stains for these digests do not reveal additional major bands (S4 Fig).

Using recombinant protein, the above assay demonstrated that the 1Q and 3Q regions, at least, are likely to be disordered. But is disorder also a hallmark of Abelson itself? As we could not isolate recombinant full length Abl from bacteria, we established lysates from third instar CNS pan-neurally expressing FLAG-tagged Abl. These lysates contain reasonable quantities of full length, as noted when we confirmed expression of our transgenes (see S1 Fig). Lysates were digested for 30 minutes at room temperature with clostripain, which has many predicted cut sites in both the globular (SH3-SH2-Kinase) domains as well as the CTD (Fig 7B). Given the use of a total lysate, the excess protein helps ensure limited digestion of Abl, and Abl digestion products could still be selectively examined by western blotting using either an anti-FLAG antibody that recognizes the C-terminal FLAG tag, or an anti-Abl antibody [64] recognizing the N-terminus including the globular SH3-SH2-kinase domain cassette. Immunoblotting with either antibody reveals that even under lysate conditions, digestion with clostripain yields a wide range of fragments consistent with disordered regions. That is, all FLAG-tagged fragments are at or below the expected size for the full-length CTD, indicating that their corresponding cut sites were within the CTD, rather than the Abl N-terminus. Conversely, immunoblotting with anti-Abl showed a series of fragments with sizes between full-length Abl and Abl N-terminus alone (69 kD), again consistent with utilization of cut sites primarily within the CTD. At higher protease concentration, a lower ~49kDa band appears that is consistent with the size of the SH3-SH2-kinase domain cassette. It is likely that the N-cap has been digested at these concentrations, and interestingly, the ANCHOR tool also predicts this region to be disordered (Fig 7A). In further support of this, the N-cap region was not resolved in the vertebrate Abl crystal structure, indicating likely disorder [65].

Together, digestion patterns with GST-1Q, 3Q and full-length Abl are consistent with high levels of disorder within the Abl CTD. Future work will have to evaluate whether they also participate in partner binding, as would be predicted based on several other studies of disordered regions [66–68].

## Discussion

**Fig 8:**
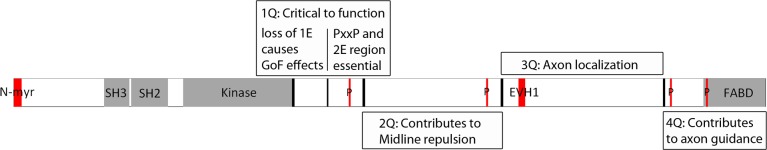
Summary schematic highlighting the role of each region of the CTD. A linear model of Abl is depicted with each quarter of the CTD delineated. The accompanying text boxes provide short statements highlighting the role that each region is thought to contribute to Abl function, as determined in our analysis. See text for details. GoF: gain-of-function.

In the three decades following Henkemeyer et al’s [28] first demonstration of the CTD’s indispensability, remarkably little headway has been made towards the understanding of the contribution of the C-terminal domain to *Drosophila* Abl function. In contrast to previous studies, we evaluated the functions of the entire length of the CTD by systematically removing large regions, to assess how this affects Abl function in axon guidance and viability. Surprisingly, Abl activity largely withstands the removal of these quarters, with the exception of the first quarter, which is absolutely essential (Fig 8). Conversely, no individual quarter (including the first) fully restores function to the N-terminus. A minimally functional Abl requires both the first and the third quarter (Abl-N-1Q-3Q), consistent with the importance of the first quarter in Abl activity and the third quarter in axon localization. However, we also uncovered subtle roles for both 2Q and 4Q in our axon guidance assays. For example, while removing 2Q mostly had no effect on Abl activity in our *Abl* rescue assays, its removal enhances midline crossover defects in a *slit* mutant, and addition of 2Q onto AblN allows it to increase the frequency of posterior commissure gaps, but not longitudinal gaps, in a *fra* mutant. Both of these results suggest that 2Q contributes specifically to midline repulsive signaling.

Similarly, 4Q also has a complex effect on Abl activity. Addition of 4Q to AblN inhibits the small amount of *Abl* axon guidance rescue seen with AblN alone, and suppresses the frequency of defects observed in *fra* mutants. Surprisingly, AblN-4Q did not alter AblN function in a *slit* mutant, although the removal of 4Q prevents ectopic crossovers in a *slit* mutant, and enhances commissure loss in a *fra* mutant. As the predominant feature of 4Q is the FABD, we hypothesize that mutations of 4Q have subtly altered the ability of Abl to regulate actin dynamics in response to receptor activation. In other work, the FABD has been linked to regulation of Abl kinase activity [31], and removal of the FABD resulted in *Abl* transgenes that were largely functional, but were subtly impaired in specific morphogenetic and midline axon guidance functions, especially when kinase activity of Abl was also removed [6, 27].

Clearly, the whole CTD contributes to Abl function, in a complex manner, likely involving different protein-protein interactions. We hypothesize that these roles are expedited by the large stretches of intrinsically disordered structure in the CTD biochemically validated in our protease assays. These disordered regions are further predicted to become ordered by gaining stabilizing energy through protein-protein interactions [61] Importantly, large spans of disorder is predicted in the CTD of both vertebrate and invertebrate Abl (see also S5 Fig) and therefore likely conserved across evolutionary history. Thus, we posit that despite divergence of primary sequence during evolution, the main function of the Abl CTD is that of a scaffolding role, much of which may be mediated by these disordered regions.

### The first quarter (1Q) plays a key regulatory role

Of our defined CTD regions, the first quarter of the CTD (1Q) is clearly the most critical region for Abl function and cannot be removed without adversely impairing function of the protein. We suspect that *AblΔ1Q* is not simply a functional null, as it also displays the most severe axon guidance phenotypes in our *fra* and *slit* gain-of-function assays, and causes significant lethality (60%) in *Abl* heterozygous animals. However, while returning 1Q by itself to AblN is sufficient to rescue adult lethality, it is relatively benign in terms of axon guidance; *AblN-1Q* fails to rescue axon guidance defects in *Abl* mutants, and does not induce gain-of-function phenotypes in *fra* or *slit* mutants. Indeed, 1Q and 3Q are minimally required together for full rescue of axon guidance, while also exhibiting few phenotypes in gain-of-function assays. The 1Q region contains a conserved sequence region, CR1 (roughly equivalent to our 2E region), previously documented by Rogers et al. [6] to reduce Abl function in many actin morphogenic events when deleted, but gain-of-function effects for this mutant were not reported. These authors postulated that the PxxP motif within CR1 carries out the key function of this region, likely through interaction of binding partners such as Crk, Nck and Abi identified in vertebrate work [40–45].

Here, we explicitly tested the role of the PxxP motif in 1Q, and also assessed whether other PxxP motifs found in 2Q and 4Q could act redundantly. Significantly, the PxxP deletion (ΔP) is the smallest mutation we made that impacts Abl function in axon guidance, and it does so in the full-length *Abl* as well as in the minimal *AblN-1Q-3Q* transgene, which lacks all other PxxP motifs. Clearly, the PxxP motif in 1Q carries out an essential function that cannot be fully replaced by the other PxxP motifs. However, the sequence context of the PxxP motif is also important. If the PxxP motif carries out the main function of 1Q, then we predict that ΔPxxP, Δ2E, and Δ1Q should impair Abl function to the same degree. Yet, *AblΔP* retains more activity than *AblΔ2E* in axon guidance rescue, and neither of these deletions replicate the severe gain-of-function effects observed when the entire 1Q is deleted. These data further suggests that, while 2E and its PxxP motif are the most important regions of 1Q, 1E must also contribute to Abl function in some contexts. This conclusion is perhaps best highlighted by the observation that the *AblN-2E-3Q* transgene, which does not have 1E, induces lethality in heterozygous animals, a feature shared only with AblΔ1Q.

Significantly, although the sequence of the 2E region is conserved when considering Drosophilids, no obvious conservation is evident between this region and the equivalent region around the PxxP motif in vertebrate Abl (Fig 1A). On the other hand, we note the presence of disorder around the PxxP motifs of both vertebrate and *Drosophila* Abl (Fig 7A and S5 Fig), and these regions have at least one common binding partner, Abi [43, 44, 69]. This suggests that conservation of this disorder may be functionally important. In other proteins, the structural plasticity of disordered regions allow them to aid in binding to partners via an induced fit mechanism[70], similar to how adjacent amino acids in other interaction motifs help determine the specificity of binding partners. Along this line it is worth pointing out that the 1E regions is also characterized by stretches of disorder, suggesting disorder maybe generally important to the function of 1Q.

The relative importance of 1Q in Abl function raises the possibility that 1Q plays a major role in regulating the scaffolding function of the entire CTD. The Crk and Nck partners identified in vertebrate work [40–42, 45] are themselves scaffold or adaptor proteins, and their recruitment through 1Q may aid in directing protein recruitment in the rest of the CTD, thus changing the output of Abl. Similarly, binding of Abi to 1Q likely recruits the SCAR/Wave complex to Abl [71, 72], and almost certainly serves as an important link between Abl and actin dynamics. It is even conceivable that these interactions regulate Abl kinase indirectly—binding of both Abi [43, 69] and Crk family proteins [73, 74] have been shown to activate Abl kinase activity. More significantly, binding of these different partners to the same PxxP motif necessitates that these interactions be mutually exclusive. Which of these interactions are most important for Abl’s role in axon guidance, as examined in our work, needs to be delineated in future studies before potential roles in regulation of Abl kinase activity become worth pursuing. Clearly, the protein-protein interactions mediated by 1Q and their effects on the scaffolding and regulatory interactions mediated by the rest of the CTD will be worth exploring.

### Axon localization

Previous work established the importance of localizing Abl to axons [27, 28, 49]. Here, we show that 3Q is the only region that is sufficient to localize a heterologous protein (RFP) to axons and is the major, although probably not sole, determinant of axon localization of Abl itself. Consistent with the role of 3Q in localization, we find that it is important for the rescue of axon guidance defects in *Abl* mutants (e.g. *AblN-1Q-3Q* vs *AblN-1Q*) and in generating gain-of-function phenotypes in *fra* or *slit* mutants (e.g. *Abl-N-3Q*).

Presently, the exact subcellular localization of Abl within the axon is unknown as we cannot distinguish between localization to the cell cortex, microtubules or axoplasm. Both RFP and AblN alone have a weak presence in axons, which we suspect represents primarily diffusion, although in the case of AblN a contribution from the SH3-SH2 domains might reinforce this localization. The axonal enrichment of Abl when 3Q is present is then likely to be due to an active process that could involve recruitment to membrane complexes, or transport down an axon in association with transport machinery and cytoskeletal elements. In epithelial cells, Abl localizes to the apical cortex, where it aids in actin morphogenic events that may involve Disabled and Ena [7, 64, 75]. However, as we could remove the EVH1 motifs within 3Q without altering axon localization, Ena binding alone is not likely to recruit 3Q to axons. We further note that RFP-2H, containing both 3Q and 4Q, localizes RFP to axons stronger than 3Q alone. Given the presence of the FABD in 4Q, we hypothesize that localization activity mediated primarily by 3Q might be stabilized by FABD-dependent interactions with actin. As the remaining regions of 3Q are characterized by intrinsic disorder, we hypothesis that axon localization is likely to be mediated by protein-protein interactions involving these disordered regions. This idea will be tested in future proteomic work.

### Intrinsic disorder and Abl function: Is Abl a hub protein?

The Abl CTD is highly susceptible to limited protease digestion, a hallmark of intrinsically disordered regions. Disordered regions are now being recognized as a major feature of ‘hub’ proteins such as BRCA1 [76], p53 [70], and myc [77, 78], all of which, like Abl, participate in diverse cellular processes. Intrinsically disordered regions provide structural plasticity ideal for participation in a variety of protein-protein interactions, including multiple simultaneous interactions or a series of individual interactions, perhaps via small linear motif elements dispersed throughout the CTD [66–68]. Certainly, the sheer size of the CTD, with most of the ~1100 amino acids predicted to be disordered, provides considerable opportunity for a diverse set of interactions. For example, in the case of p53, a major hub protein associated with dozens of targets, just over two-thirds of its protein interactions are mediated by the disordered N- and C-terminal regions [70]. In some cases, competition between binding partners for a particular interface is observed, a phenomenon that may be occurring in the 2E region if the disorder surrounding the PxxP motif helps dictate binding partners (Crk, Nck or Abi). On the other hand, p53 also appears to use larger stretches of disorder to bind to several partners simultaneously [70]. In this case, the various disordered regions of the Abl CTD would be expected to display a level of promiscuity, binding to many partners to form dynamic, heterogeneous ‘fuzzy’ complexes [79]. This could in part explain why our deletion transgenes often display differential effects between the *Abl*, *fra* and *slit* genetic backgrounds, especially with regard to the subtle additive effects observed for 2Q mutants in our *slit* and *fra* gain-of-function assays.

In conclusion, given the parallels to p53, we propose that Abl functions as a hub protein, using the intrinsic disorder of its CTD to participate in a variety of protein-protein interactions underlying Abl’s myriad of cell functions. Minimally, if it is indeed disorder, rather than specific sequences, which is conserved in the CTD, and govern function, future examination of Abl will have to move away from a focus on single motifs and towards identifying protein partners participating in signaling or scaffolding mediated by larger regions of the CTD.

## Materials & methods

### Bioinformatics

Amino acid sequences for Abl homologs were retrieved from the Ensembl and EnsemblGenomes databases [80, 81] (see S2 and S3 Tables for full set of homologs used in alignment). Multiple sequence alignments were carried out via Clustal Omega [82, 83], and the evolutionary conservation of amino acid sequences were estimated using the ConSurf web server in sequence-only mode [84–87]. Protein domains in Abl homologs were annotated using SMART [88, 89], and motifs and other features were manually annotated. Intrinsic disorder predictions were carried out using the IUPred web server for long disorder prediction [59, 90], and predictions of protein binding regions within disordered regions were carried out using the ANCHOR web server [61, 62].

### Fly genetics and stocks

Flies were cultured at 25°C on standard cornmeal-molasses medium on a 12 hour light/dark cycle. The following alleles were used: *Abl*^*4*^, *Abl*^*2*^, *fra*^*4*^, *fra*^*3*^, *slit*^*2*^, *Su(P)*^*EY13245*^, and *UAS-mCD8-GFP*. The following Gal4 drivers were used: *insc*^*Mz1407*^ *(1407-Gal4)*, *Act5C-Gal4*, *elav-Gal4*, *65D01-Gal4*, and *60H11-Gal4*. *UAS-Abl* transgene stocks are as further described below. Stocks were balanced over LacZ-containing balancer chromosomes where appropriate for detection of embryo genotypes. Other fly stocks were obtained from Bloomington Drosophila Stock Center.

### Transgenic constructs

For Abl transgenes with deletions of 1Q, 2Q, 3Q, 4Q or ΔP, Δ1E and Δ2E mutations, Abl cDNA was subcloned from a pMT vector [14] into the pUASTattB vector [46] for PhiC31-mediated transgenesis. Deletions of CTD quarters in this construct were then carried out via PCR site-directed mutagenesis, with the insertion of an AgeI site at the joints. The ΔP, Δ1E and Δ2E mutations were made via the Phusion site-directed mutagenesis method with blunt end-joining. For transgenes containing Abl CTD fragments joined to either RFP or AblN, RFP and AblN constructs were first made in the pUASTattB vector, containing either BsrGI/NotI or AgeI/NotI sites at their C-termini for insertion of CTD fragments. Fragments were then amplified with the appropriate sites and inserted. The AblN-1Q transgene was amplified as a single fragment from the 5’ end of the Abl N-terminus to the end of the 1Q region, then inserted into the pUASTattB vector. Primer sequences are found in S4 Table.

Transgenic constructs containing multiple deletions were carried out either via multiple rounds of site-directed mutagenesis, or by assembly of fragments from constructs containing single deletions. All constructs were verified by sequencing. Transgenic flies were generated by germline transformation into fly stocks containing the *M{vas-int*.*Dm}ZH-2A* maternal integrase source and an attP landing site. The *ZH-attP-86Fb* landing site was used for RFP transgenes, while the *ZH-attP-22A* landing was used for Abl transgenes [46]. All fly germline transformations were carried out by Rainbow Transgenic Flies, Inc.

Expression levels of *Abl* transgenes were compared by dissecting 5 CNS’s from 3^rd^ instar larvae expressing said transgenes with *1407-Gal4*, following by lysis in SDS sample buffer. Anti-FLAG western blots were carried out with 1:10000 monoclonal rat anti-FLAG L5 (Thermo Fisher, Cat# MA1-142-1MG, Lot# QB215988) followed by 1:20000 polyclonal HRP-conjugated Goat anti-rat antibody (Jackson Immunoresearch, Cat# 112-035-003). Blots were then reprobed with 1:500 monoclonal mouse anti-β-tubulin (DSHB, Cat#E7, Lot# 1/30/14) followed by 1:20000 polyclonal HRP-conjugated goat anti-mouse antibody (Jackson Immunotech, Cat# 115-035-003, Lot# 120344). Dissections and western blots were repeated 3 times.

### Embryo collection, immunohistochemistry, and immunofluorescence

Embryos were collected by standard techniques as previously detailed [14]. Briefly, flies were allowed to mate and lay embryos overnight on apple juice agar. Embryos were collected and dechorionated with 25% bleach, then fixed in formaldehyde-saturated heptane for 45 min. Vitelline membranes were cracked by addition of methanol to embryos in heptane followed by vigorous shaking and further washes in methanol, then rehydrated in PBT (1x PBS, 0.1% Triton X-100). To mark embryos carrying LacZ balancer chromosomes, embryos were then incubated in X-gal staining solution (1x PBS, 2 mM MgCl_2_, 5 mM potassium ferrocyanide, 5 mM potassium ferricyanide, 4 μL/mL 20% X-gal in dimethylformamide) for 1–3 hours. Embryos were then post-fixed in 4% formaldehyde in PBS for 10 min.

For immunohistochemistry, embryos were incubated with either monoclonal antibody 1D4 (DSHB, Cat# 1D4 anti-Fas II-s, Lot# 12/3/09) or BP102 (DSHB, Cat# BP 102 anti-CNS axons-s, Lot# 07/Apr/10) at a 1:10 dilution in PBT + 5% normal goat serum for 4 hours or overnight. Secondary polyclonal HRP-conjugated goat anti-mouse antibody (Jackson Immunotech, Cat# 115-035-003, Lot# 120344) was used at a 1:500 dilution with incubation for 4 hours or overnight. Chromogenic detection was carried out by incubation of embryos in Stable DAB solution (Thermo Fisher, Cat# 750118) for a few minutes until appropriately intense staining was observed. Embryos were then washed with PBT and cleared in 70% glycerol. Imaging was carried out on a Leica DM5500 B microscope with differential interference contrast optics and a HCX PL APO 100x/1.40–0.70 OIL objective at room temperature. Images were captured with a Leica DFC425C color camera using the LAS AF acquisition software.

For immunofluorescence to determine localization of transgenic Abl proteins, devitellinized embryos fixed in formaldehyde-saturated heptane were not subject to X-gal staining but were used directly for staining. Embryos were incubated overnight for each of the following antibodies, with washes with PBT between antibodies: 3:2000 monoclonal rat anti-FLAG L5 (Thermo Fisher, Cat# MA1-142-1MG, Lot# QB215988), 3:1000 polyclonal Dylight 594-conjugated goat anti-rat (Thermo Fisher, Cat# SA5-10020, Lot# RF2218502), 1:5 mAb BP102 (DSHB, Cat# BP 102 anti-CNS axons-s, Lot# 07/Apr/10), 3:2000 polyconal Alexa 488-conjugated Goat anti-mouse (Jackson Immunotech, Cat# 115-545-166, Lot# 128727). Embryos were then counterstained with 1 μg/mL DAPI dihydrochloride in PBS (Santa Cruz Biotechnology, Cat# sc-3598) for 20 minutes, and then cleared in 90% glycerol.

### Embryonic phenotype quantification

Embryos were sorted by developmental stages as previously defined [91], and were counted by a trained scorer blinded to embryo genotypes. Scoring was done on a Leica DM5500B microscope at 1000x magnification with brightfield and differential interference contrast optics.

For rescue of *Abl* midline crossover defects and enhancement of *slit* midline crossover defects, whole mount embryos of stages 16–17, stained for mAb 1D4 were analyzed. All abdominal and thoracic segments were evaluated for each embryo. Ectopic crossing overs were defined as Fas2-positive axon bundles crossing the midline and joining the Fas2-positive fascicles on the opposite side. Three replicates per genotype were counted, with about 30 embryos per replicate.

For analysis of guidance defects in *fra* mutants, whole mount embryos of stages 15–17 stained for mAb BP102 were analyzed. All abdominal and thoracic segments were evaluated for each embryo. Commissures were analyzed on a per-segment basis, while longitudinal connectives were analyzed at the intersegmental region. Thinning of commissures or longitudinal connectives was defined as a moderate but visible reduction in thickness of BP102-positive axons in that region, while gaps were defined as a complete or near-complete loss of BP102-positive axons.

### Imaging and quantification of Abl localization

For quantification of Abl localization, embryonic nerve cords of filleted stage 17 embryos were mounted in Vectashield antifade reagent (Vector labs, Cat#H-1000) and imaged using the Leica TCS SP5 point scanning confocal system attached to the Leica DMI6000 CS inverted microscope at room temperature using a HC PL APO 100x/1.44 OIL CORR CS objective, with the LAS X image acquisition software. Images were acquired as a confocal stack with 0.3 μm slices such as to span the vertical width of the axon tracts of the nerve cord. Confocal microscopy was done at the Microscopy, Imaging and Cytometry Resources Core at Wayne State University, School of Medicine. Quantification of localization was done using the Fiji software [92, 93]. To distinguish between areas of axon tracts and cell bodies, stacks were flattened, and any area outside the nerve cord was first manually excluded from analysis. Fluorescent signal in the green channel, stained for the BP102 axonal marker, was thresholded using the minimum cross entropy method [94] built into Fiji. The thresholded channel was then used as a mask to select the area corresponding to axon tracts in the red anti-FLAG channel, while the inverse selection of this area was defined as the cell bodies. The mean intensity of FLAG staining was measured in these two areas, and background levels of fluorescence were subtracted using equivalent measurements from the average of 3 non-expressing embryos. The ratio of intensity in axon tracts vs cell bodies were taken as a measure of Abl localization. Measurements were taken for 3 replicate nerve cords per transgene.

### Adult lethality counts

In scoring rescue, it is important to establish a category of flies from the same cross that allows us to determine the number of progeny we might expect if fully rescued. This category of progeny consists of flies that are expected to fully eclose at a wild-type rate, and we use *Abl* heterozygous flies that do not express any transgene for this purpose. For *Abl* adult lethality rescue usingUAS-Abl transgenes, the following schemes were used. For the *Act5C-Gal4* and 3^rd^ chromosome *Abl-Gal4* lines, R60H11 and R65D01 [50], *UAS-Abl; Abl*^*2*^ *e/TM3*, *Sb Act-LacZ* females were crossed to *Gal4 Abl*^*4*^*/+* males. The R60H11 and R65D01 drivers were selected out of a larger collection of *Abl-Gal4* lines constructed with various *Abl* enhancer fragments, based on their relatively widespread expression in a large subset of CNS neurons (see S3 Fig for expression patterns). Rescued *Abl* homozygous flies (*w; UAS-Abl/+; Gal4 Abl*^*4*^*/Abl*^*2*^ *e*) were normalized to non-expressing, *Abl*^*2*^ heterozygous flies (*w; UAS-Abl/+; Abl*^*2*^ *e/+*). Three replicates of ~200 flies each were counted.

For rescue with the *1407-Gal4* driver, the following scheme was used:

$$y\;w;\;UAS\text{-}Abl;\;Abl^{2}/TM3,\;Sb\;Act\text{-}LacZ\;X\;y\;w;\;1407\text{-}Gal4/+;\;Abl^{4}/Su{\left(P\right)}^{EY13245}$$

The *Su(P)*^*EY13245*^ allele is a homozygous viable P-element insertion adjacent to the Abl gene, that carries both mini-white and mini-yellow markers. The mini-yellow marker allows us to distinguish Abl heterozygous flies (*Abl*^*2*^*/Su(P)*^*EY13245*^) from Abl homozygous flies. Flies carrying the *1407-Gal4* insertion were further distinguished by intensity of the mini-white eye color. Rescued flies (*y w; 1407-Gal4/UAS-Abl; Abl*^*4*^*/Abl*^*2*^) were normalized to *Abl* heterozygous flies not expressing the transgene (*y w; UAS-Abl/+; Abl*^*2*^*/ Su(P)*^*EY13245*^). Three replicates of ~400 flies each were counted.

### GST constructs and E. coli expression

The pGEX-6P-1 vector was modified by site-directed mutagenesis to include AgeI and PmeI sites in the multicloning site. 1Q and 3Q were cloned as a FLAG-tagged AgeI/PmeI fragment into said vector. GST-1Q and 3Q were expressed in the *E*. *coli* BL21 strain at 18°C, and purified on Glutathione Sepharose 4B (GE Life Science, Cat#17075601) and eluted with reduced glutathione. Excess glutathione was dialyzed before further use.

### Protease susceptibility assay

Digestion was carried out in digestion buffer consisting of 50 mM Tris pH 7.4, 150 mM NaCl, 1 mM CaCl_2_, 1 mM 2-mercaptoethanol and 1% IGEPAL-CA630 (Sigma-Aldrich, Cat# I8896). For digestion of GST-1Q and -3Q, a range of 6 clostripain (Worthington Biochemical Corporation, Cat# LS001641) concentrations ranging from 2 x 10^−1^ to 6.25 x 10^−3^ μg/mL (doubling dilutions) was set up in 100 μL volume of digestion buffer. Clostripain concentrations of 0, 2 (10x) and 20 (100x) μg/mL were also set up. Following that, ~5 μg purified GST-1Q or -3Q in 20 μL buffer was added to each digest, and digestion was carried out for 30 min at room temperature. Digests were stopped with 2x SDS loading buffer containing 5 mM EDTA and 1mM PMSF, followed by 5 minutes incubation at 95°C. Anti-FLAG western blots were carried out with 1:10000 monoclonal rat anti-FLAG L5 (Thermo Fisher, Cat# MA1-142-1MG, Lot# QB215988) followed by 1:20000 HRP-conjugated polyclonal Goat anti-rat antibody (Jackson Immunoresearch, Cat# 112-035-003). Anti-GST western blots were carried out with 1:5000 polyclonal rabbit anti-GST (FisherSci, Cat# CAB4169, Lot# QJ210646) followed by 1:10000 polyclonal HRP-conjugated Goat anti-rabbit antibody (Jackson Immunoresearch, Cat# 111-035-144). Digestions were repeated 3 times.

For digestion of full-length Abl, the wild-type *UAS-Abl* transgene was expressed in 3^rd^ instar larvae with *1407-Gal4*. The CNS of 10 third instars were dissected, pooled and frozen. CNSes were lysed in 200 μL of 50 mM Tris pH 7.4, 150 mM NaCl, 1% IGEPAL-CA630, 0.5x ProBlock Gold Protease Inhibitor Cocktail (Gold Biotechnology, Cat# GB-108-2), 1 mM EDTA by 20 strokes of a Dounce homogenize with pestle B. Lysate was cleared by centrifugation, and was used immediately for clostripain digestion. A range of 8 clostripain concentrations ranging from 2 to 1.563 x 10^−3^ μg/mL (doubling dilutions), as well as a zero control, were set up in 100 μL volumes. 20 μL lysate was added to each digest, and digestions were carried out as for GST-1Q and -3Q. Anti-FLAG western blots were carried out as for GST-1Q and -3Q. Anti-Abl western blots were carried out with 1:5000 polyclonal rabbit anti-Abl [64] (a kind gift from Dr. Edward Giniger) followed by 1:10000 polyclonal HRP-conjugated Goat anti-rabbit antibody (Jackson Immunoresearch, Cat# 111-035-144). Digestions were repeated 3 times.

### Statistical analyses

All statistical analyses were carried out in R version 3.3.1 with the multcomp [95], MASS [96], ggplot2 [97], car [98], and DHARMa [99] packages.

For axon guidance defect counts in embryos in the *Abl*, *fra* or *slit* genetic backgrounds, data from all 3 replicates for each separate dataset (*Abl*, *fra* or *slit*) were pooled and fitted to negative binomial generalized linear models, with number of defects per embryo as the response variable and transgene expressed as the predictor variable. Appropriateness of the negative binomial distribution over the poisson distribution was determined using the dispersion test [100–102] to estimate overdispersion. Scaled residual plots were produced using the DHARMa package for diagnostics, and examined manually.

For adult lethality counts normalized rescued fly counts were transformed by the square root transformation. Homogeneity of variances of the transformed values was determined by Levene’s test from the car package, and data was then fitted to an ANOVA model with the square root of normalized rescued fly counts as the response variable and transgene expressed as the predictor variable. Diagnostic plots were produced using the ggplot2 package, and manually examined. Post-hoc analysis was carried out with Dunnett’s test from the multcomp package, and comparisons were made between the entire transgene set and the *Abl*^*4*^*/Abl*^*2*^, *AblWT*, and *AblN* treatments.

For quantification of Abl transgenic protein localization, ratios of axon to cell body intensity were transformed by the log_10_ transformation to fit assumptions of normality. Homogeneity of variances of the transformed values was determined by Bartlett’s test [103], and data was then fitted to a one-way ANOVA model with log_10_ ratio of axon to cell body intensity as the response variable and transgene expressed as the predictor variable. Diagnostic plots were plotted with R utilizing the ggplot2 package, and examined manually. Post-hoc analysis was carried out with Dunnett’s test from the multcomp package. For the Abl transgene dataset, comparisons were made between the entire transgene set and the AblN and AblWT transgenes individually. For the RFP transgene dataset, comparisons were made between the entire transgene set and the RFP and RFP-AblCTD transgenes, individually.

## Supporting information

S1 Fig Expression levels of *Abl* transgenes generated in this work. Transgenes were expressing in 3^rd^ instar CNS’s with *1407-Gal4*, and 5 CNS’s were dissected and lysed in SDS sample buffer. Western blots shown are representative of three replicates.
(TIF)

S2 Fig Adult rescue by Abl CTD mutant transgenes. Asterisks (*) over a particular column indicate significant difference (p < 0.05) compared to *Abl*^*4*^*/Abl*^*2*^ homozygous embryos. (A) *Abl* transgenes were ubiquitously expressed using the *Actin5C-Gal4* driver and the percent of expected adults eclosing of either sex is quantified. Three replicates of approximately 200 flies were counted, with at least 50 flies in the non-expressing *Abl* heterozygous category. Only *AblΔ4Q*, *AblN-1Q*, *AblN-1Q-3Q* and *AblCTD* show significant rescue. Mutants that have 1E, 2E or P removed have small effects on rescue. (B) Quantification of *Abl*^*4*^*/+* heterozygote survival when *Abl* transgenes are pan-neurally expressed with *1407-Gal4*. These counts are from the same dataset as Fig 3A. Only *AblΔ1Q* and *AblN-2E-3Q* cause significant lethality compared to non-expressing *Abl* heterozygotes in this condition. (C) Rescue for males with the *1407-Gal4*, *65D01-Gal4* and *60H11-Gal4* drivers. For the *65D01-Gal4* and *60H11-Gal4* drivers, three replicates of approximately 200 flies were counted, with approximately 50 flies in the non-expressing Abl heterozygous category, used to normalize rescued Abl mutant flies. Only males are shown for comparison purposes as the *65D01-Gal4* and *60H11-Gal4* drivers give poor rescue for females. Significance indicated by symbols as defined in legend top right corner.
(TIF)

S3 Fig Expression pattern of the *1407-Gal4, 65D01-Gal4* and *60H11-Gal4* drivers in embryos and larval CNS. The indicated drivers were used to express *UAS-mCD8-GFP*, and embryos/larvae were immunostained for GFP. Stage 13/17 whole mount embryos and 3^rd^ instar larval CNS are shown for *1407-Gal4* (A), *65D01-Gal4* (B) and *60H11-Gal4* (C).
(TIF)

S4 Fig **Total protein stain for clostripain digests of GST-1Q (A) and -3Q (B).** Digests from Fig 7 were stained for the total protein stain Sypro Ruby. The symbols ▻GST-1Q and ▻GST-1Q indicate expected sizes for full-length protein. ►C indicates expected bands originating from added clostripain.
(TIF)

S5 Fig Bioinformatic analysis of two representative vertebrate Abl homologs. The following sections apply to both murine ABL1 (A) and ABL2/ARG (B): (i) Presence of domains as annotated by SMART (grey) and motifs (red). Nuclear localization and export signals, and additional functional regions are as previously defined [104–108]: high mobility group 1-like boxes (HLB, in pink), F-actin binding region (FAB, in teal) and microtubule-binding region (MT, in purple). (ii) The CTD of both murine ABL1 and ABL2/ARG are intrinsically disordered as predicted by IUPred. (iii) Both CTDs have disordered regions that may function in protein binding as predicted by ANCHOR.
(TIF)

S1 Table Quantification of various axon guidance defects caused by overexpression of *Abl* transgenes in a *fra* homozygous background. Shown here are per-embryo defect counts for anterior commissure thinning and gaps, posterior commissure thinning and longitudinal connective thinning. Posterior commissure gaps and longitudinal connective gaps are shown in Fig 5.
(PDF)

S2 Table Gene names and Uniprot accession numbers of Drosophilid *Abl* homologs used for multiple sequence alignment. (PDF)

S3 Table Gene names and Uniprot accession numbers of vertebrate and invertebrate *Abl* homologs used for multiple sequence alignment. (PDF)

S4 Table Primers used in the generation of *Abl* mutant transgenes in this work. (PDF)
