# Supplementary figures and images for: Long disordered regions of the C-terminal domain of Abelson tyrosine kinase have specific and additive functions in regulation and axon localization
Source: PLoS One. 2017 Dec 12;12(12):e0189338. doi: 10.1371/journal.pone.0189338 (PMC5726718; doi:10.1371/journal.pone.0189338)

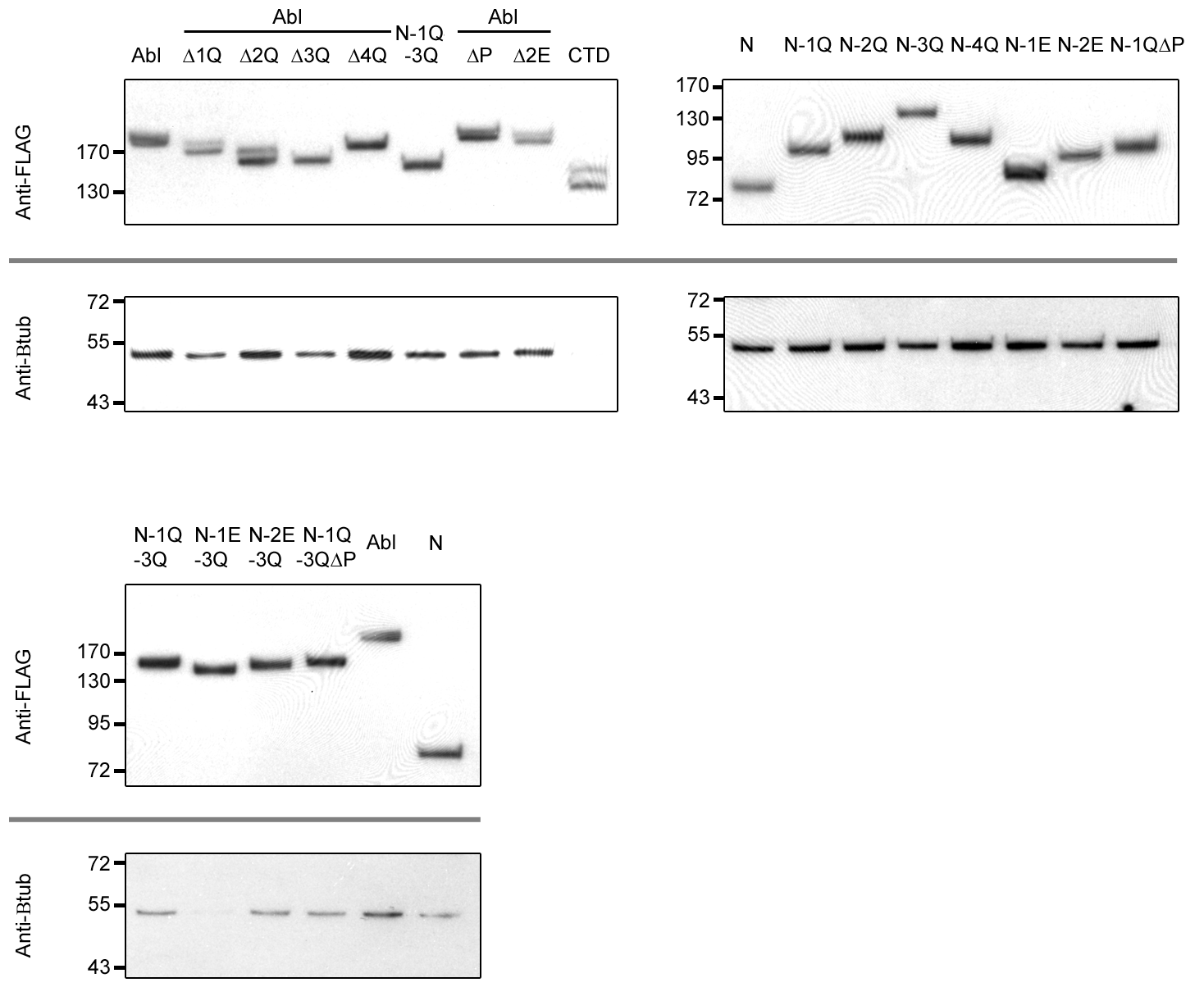

Supplement: S1 Fig — Transgenes were expressing in 3rd instar CNS’s with 1407-Gal4, and 5 CNS’s were dissected and lysed in SDS sample buffer. Western blots shown are representative of three replicates. (TIF) [file pone.0189338.s001.tif]

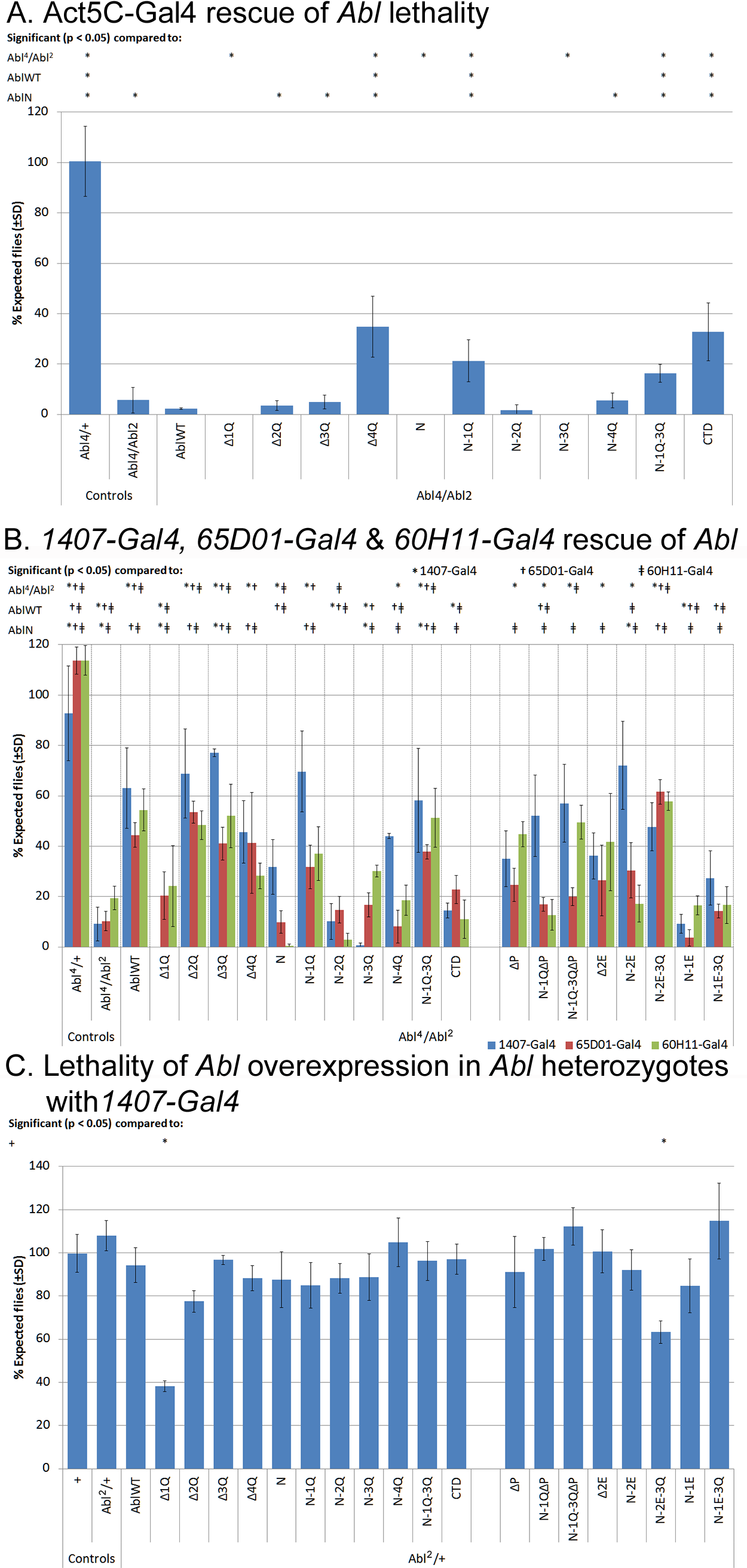

Supplement: S2 Fig — Asterisks (*) over a particular column indicate significant difference (p < 0.05) compared to Abl4/Abl2 homozygous embryos. (A) Abl transgenes were ubiquitously expressed using the Actin5C-Gal4 driver and the percent of expected adults eclosing of either sex is quantified. Three replicates of approximately 200 flies were counted, with at least 50 flies in the non-expressing Abl heterozygous category. Only AblΔ4Q, AblN-1Q, AblN-1Q-3Q and AblCTD show significant rescue. Mutants that have 1E, 2E or P removed have small effects on rescue. (B) Quantification of Abl4/+ heterozygote survival when Abl transgenes are pan-neurally expressed with 1407-Gal4. These counts are from the same dataset as Fig 3A. Only AblΔ1Q and AblN-2E-3Q cause significant lethality compared to non-expressing Abl heterozygotes in this condition. (C) Rescue for males with the 1407-Gal4, 65D01-Gal4 and 60H11-Gal4 drivers. For the 65D01-Gal4 and 60H11-Gal4 drivers, three replicates of approximately 200 flies were counted, with approximately 50 flies in the non-expressing Abl heterozygous category, used to normalize rescued Abl mutant flies. Only males are shown for comparison purposes as the 65D01-Gal4 and 60H11-Gal4 drivers give poor rescue for females. Significance indicated by symbols as defined in legend top right corner. (TIF) [file pone.0189338.s002.tif]

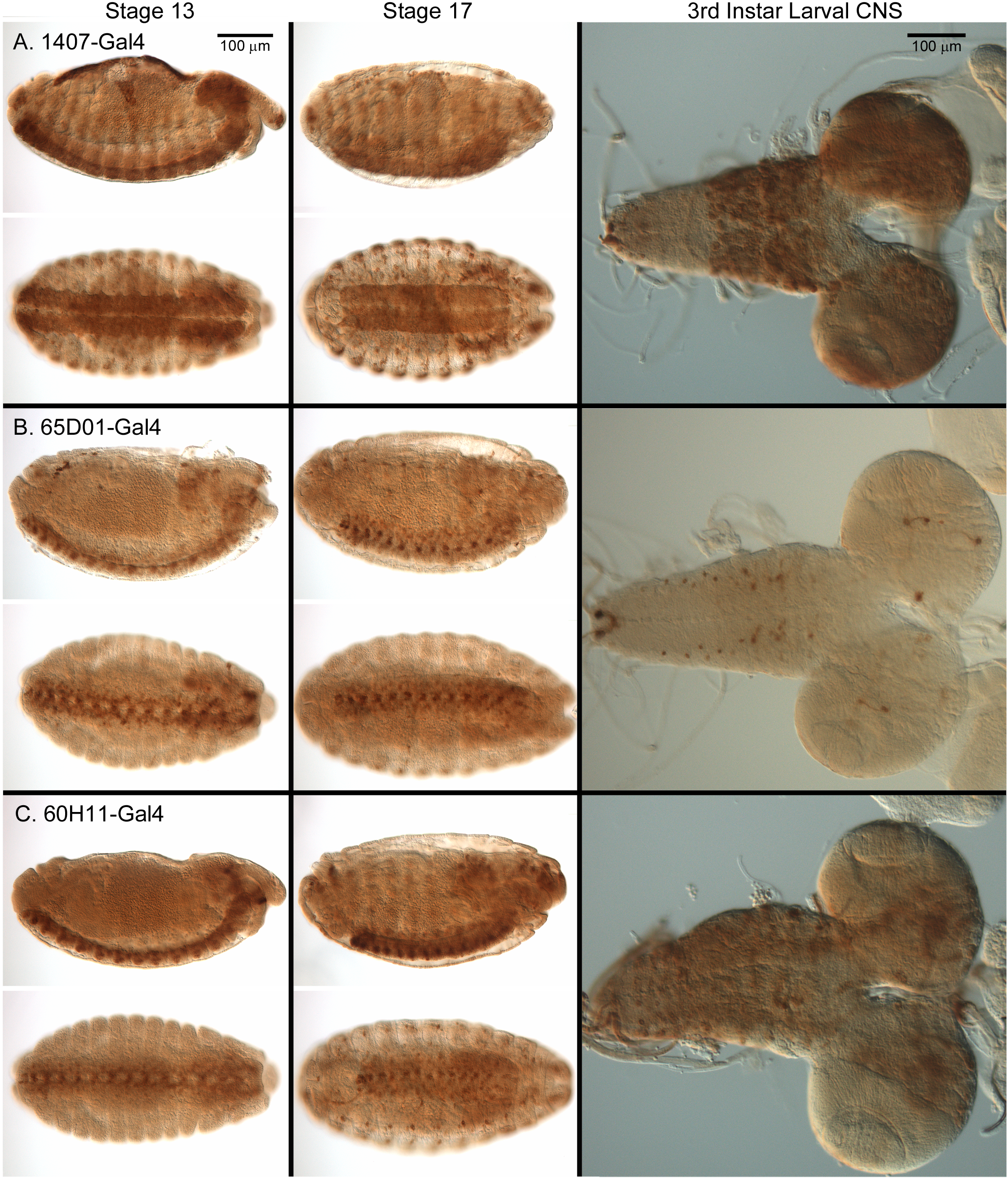

Supplement: S3 Fig — The indicated drivers were used to express UAS-mCD8-GFP, and embryos/larvae were immunostained for GFP. Stage 13/17 whole mount embryos and 3rd instar larval CNS are shown for 1407-Gal4 (A), 65D01-Gal4 (B) and 60H11-Gal4 (C). (TIF) [file pone.0189338.s003.tif]

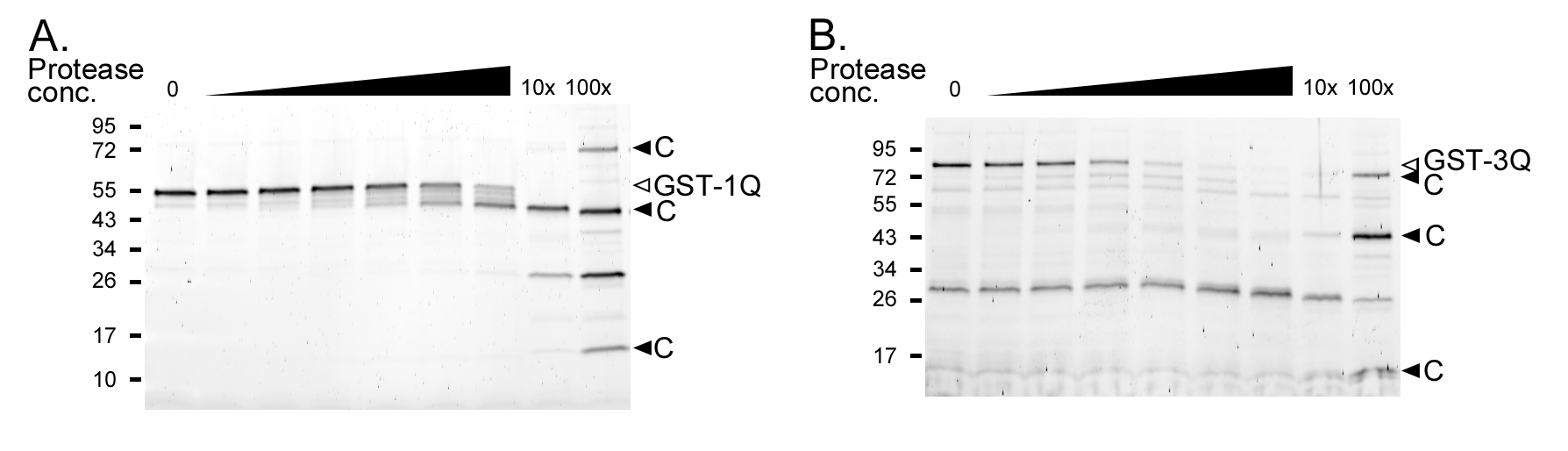

Supplement: S4 Fig — Total protein stain for clostripain digests of GST-1Q (A) and -3Q (B). Digests from Fig 7 were stained for the total protein stain Sypro Ruby. The symbols ▻GST-1Q and ▻GST-1Q indicate expected sizes for full-length protein. ►C indicates expected bands originating from added clostripain. (TIF) [file pone.0189338.s004.tif]

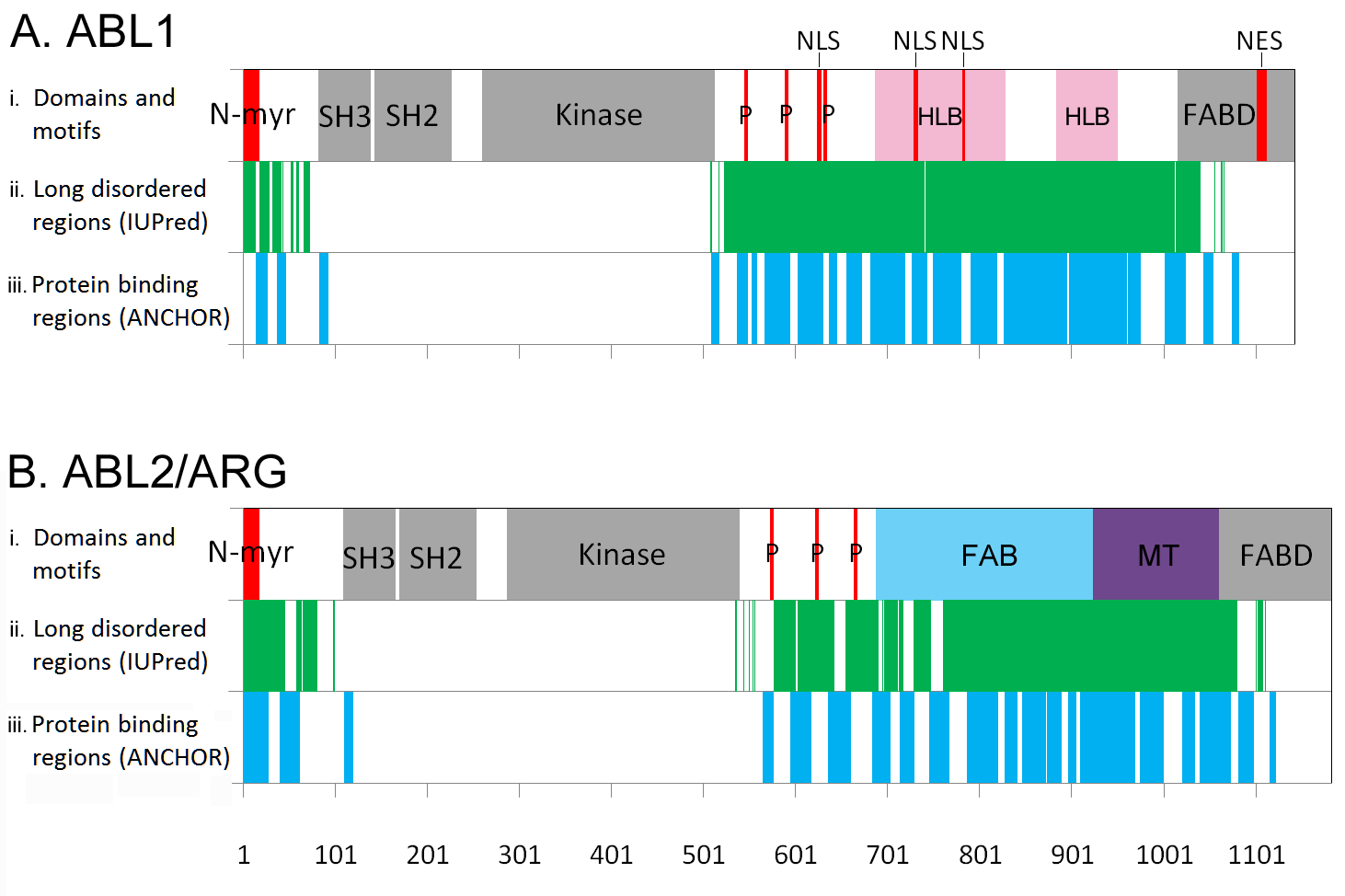

Supplement: S5 Fig — The following sections apply to both murine ABL1 (A) and ABL2/ARG (B): (i) Presence of domains as annotated by SMART (grey) and motifs (red). Nuclear localization and export signals, and additional functional regions are as previously defined [104–108]: high mobility group 1-like boxes (HLB, in pink), F-actin binding region (FAB, in teal) and microtubule-binding region (MT, in purple). (ii) The CTD of both murine ABL1 and ABL2/ARG are intrinsically disordered as predicted by IUPred. (iii) Both CTDs have disordered regions that may function in protein binding as predicted by ANCHOR. (TIF) [file pone.0189338.s005.tif]
