# Supplementary material for: Long disordered regions of the C-terminal domain of Abelson tyrosine kinase have specific and additive functions in regulation and axon localization
Source: PLoS One. 2017 Dec 12;12(12):e0189338. doi: 10.1371/journal.pone.0189338 (PMC5726718; doi:10.1371/journal.pone.0189338)
Supplement: S1 Table — Shown here are per-embryo defect counts for anterior commissure thinning and gaps, posterior commissure thinning and longitudinal connective thinning. Posterior commissure gaps and longitudinal connective gaps are shown in Fig 5. (PDF) [file pone.0189338.s006.pdf]

S1 Table. Quantification of various axon guidance defects caused by overexpression of Abl transgenes in a fra homozygous background.

		Anterior commissure thinning (%)						Anterior commissure gaps (%)					
Transgene	Embryo count	0	1	2	3	4	≥5	0	1	2	3	4	≥5
RFP	177	93.2	6.8	0.0	0.0	0.0	0.0	97.2	2.8	0.0	0.0	0.0	0.0
AblWT	182	88.5	9.9	1.6	0.0	0.0	0.0	98.9	1.1	0.0	0.0	0.0	0.0
Δ1Q	174	71.8	20.1	5.2	2.9	0.0	0.0	96.6	2.9	0.6	0.0	0.0	0.0
Δ2Q	90	91.1	8.9	0.0	0.0	0.0	0.0	98.9	1.1	0.0	0.0	0.0	0.0
Δ3Q	69	95.7	4.3	0.0	0.0	0.0	0.0	98.6	1.4	0.0	0.0	0.0	0.0
Δ4Q	75	84.0	12.0	2.7	1.3	0.0	0.0	98.7	1.3	0.0	0.0	0.0	0.0
N	181	88.4	10.5	0.6	0.6	0.0	0.0	97.8	2.2	0.0	0.0	0.0	0.0
N-1Q	189	87.8	11.1	0.5	0.0	0.5	0.0	94.7	4.2	0.5	0.0	0.0	0.5
N-2Q	88	84.1	12.5	3.4	0.0	0.0	0.0	98.9	1.1	0.0	0.0	0.0	0.0
N-3Q	86	79.1	15.1	3.5	1.2	0.0	1.2	95.3	3.5	0.0	0.0	0.0	1.2
N-4Q	80	93.8	6.3	0.0	0.0	0.0	0.0	98.8	1.3	0.0	0.0	0.0	0.0
N-1Q-3Q	55	89.1	9.1	0.0	1.8	0.0	0.0	98.2	1.8	0.0	0.0	0.0	0.0
CTD	69	94.2	5.8	0.0	0.0	0.0	0.0	98.6	1.4	0.0	0.0	0.0	0.0
ΔP	95	83.2	15.8	1.1	0.0	0.0	0.0	98.9	1.1	0.0	0.0	0.0	0.0
N-1QΔP	90	86.7	12.2	0.0	1.1	0.0	0.0	94.4	5.6	0.0	0.0	0.0	0.0
N-1Q-3QΔP	91	84.6	12.1	3.3	0.0	0.0	0.0	95.6	4.4	0.0	0.0	0.0	0.0
Δ2E	87	80.5	10.3	8.0	1.1	0.0	0.0	90.8	9.2	0.0	0.0	0.0	0.0
N-2E-3Q	94	81.9	12.8	3.2	1.1	0.0	1.1	94.7	5.3	0.0	0.0	0.0	0.0
N-1E-3Q	89	80.9	15.7	3.4	0.0	0.0	0.0	95.5	4.5	0.0	0.0	0.0	0.0

		Posterior commissure thinning (%)						Longitudinal connective thinning (%)					
Transgene	Embryo count	0	1	2	3	4	≥5	0	1	2	3	4	≥5
RFP	177	16.9	34.5	26.6	11.9	5.6	4.5	15.8	26.6	23.7	22.0	7.3	4.5
AblWT	182	12.1	18.1	23.1	24.7	14.3	7.7	8.2	12.1	25.8	18.7	19.2	15.9
Δ1Q	174	4.0	6.9	23.0	24.1	23.0	19.0	6.9	12.1	10.9	26.4	21.8	21.8
Δ2Q	90	17.8	27.8	28.9	11.1	13.3	1.1	2.2	6.7	13.3	21.1	23.3	33.3
Δ3Q	69	14.5	15.9	40.6	17.4	4.3	7.2	14.5	27.5	17.4	24.6	7.2	8.7
Δ4Q	75	8.0	14.7	18.7	18.7	24.0	16.0	2.7	1.3	18.7	36.0	16.0	25.3
N	181	11.0	27.1	25.4	21.5	9.9	5.0	11.6	17.7	25.4	24.3	12.7	8.3
N-1Q	189	15.9	25.9	20.1	18.5	11.6	7.9	19.0	24.3	25.9	19.0	5.3	6.3
N-2Q	88	5.7	6.8	26.1	26.1	20.5	14.8	6.8	10.2	25.0	22.7	21.6	13.6
N-3Q	86	1.2	17.4	17.4	24.4	18.6	20.9	4.7	8.1	15.1	29.1	12.8	30.2
N-4Q	80	26.3	31.3	15.0	13.8	5.0	8.8	16.3	13.8	26.3	16.3	12.5	15.0
N-1Q-3Q	55	14.5	32.7	20.0	16.4	10.9	5.5	0.0	5.5	14.5	25.5	23.6	30.9
CTD	69	37.7	21.7	30.4	7.2	2.9	0.0	15.9	18.8	23.2	27.5	7.2	7.2
ΔP	95	3.2	18.9	28.4	22.1	14.7	12.6	10.5	37.9	20.0	16.8	10.5	4.2
N-1QΔP	90	12.2	26.7	30.0	14.4	7.8	8.9	18.9	23.3	28.9	21.1	6.7	1.1
N-1Q-3QΔP	91	15.4	31.9	23.1	14.3	11.0	4.4	17.6	35.2	20.9	16.5	6.6	3.3
Δ2E	87	8.0	37.9	17.2	19.5	9.2	8.0	23.0	28.7	20.7	13.8	8.0	5.7
N-2E-3Q	94	7.4	18.1	25.5	20.2	11.7	17.0	12.8	25.5	19.1	22.3	13.8	6.4
N-1E-3Q	89	9.0	18.0	29.2	23.6	13.5	6.7	28.1	22.5	22.5	13.5	7.9	5.6

Shown here are per-embryo defect counts for anterior commissure thinning and gaps, posterior commissure thinning and longitudinal connective thinning. Posterior commissure gaps and longitudinal connective gaps are shown in figure 5.
