# Supplementary material for: Long disordered regions of the C-terminal domain of Abelson tyrosine kinase have specific and additive functions in regulation and axon localization
Source: PLoS One. 2017 Dec 12;12(12):e0189338. doi: 10.1371/journal.pone.0189338 (PMC5726718; doi:10.1371/journal.pone.0189338)
Supplement: S2 Table — (PDF) [file pone.0189338.s007.pdf]

S2 Table. Gene names and Uniprot accession numbers of Drosophilid Abl homologs used for multiple sequence alignment.

**Species**	**Gene name**	**Uniprot accession #**
Drosophila melanogaster (Fruit fly)	*Abl*	M9PFS1
Drosophila mojavensis (Fruit fly)	*Dmoj\GI16710*	B4L9R9
Drosophila virilis (Fruit fly)	*Dvir\GJ12453*	B4LCY8
Drosophila grimshawi (Fruit fly)	*Dgri\GH16452*	B4J010
Drosophila willistoni (Fruit fly)	*Dwil\GK20085*	B4MXF9
Drosophila pseudoobscura pseudoobscura (Fruit fly)	*Dpse\GA17894*	Q29D50
Drosophila ananassae (Fruit fly)	*Dana\GF10512*	B3M4F6
Drosophila sechellia (Fruit fly)	*Dsec\GM24379*	B4HJR1
Drosophila simulans (Fruit fly)	*Dsim\GD12452*	B4QMW5
Drosophila erecta (Fruit fly)	*Dere\GG15861*	B3NDJ4
Drosophila yakuba (Fruit fly)	*Dyak\GE22200*	B4PKB7
