# Supplementary material for: Long disordered regions of the C-terminal domain of Abelson tyrosine kinase have specific and additive functions in regulation and axon localization
Source: PLoS One. 2017 Dec 12;12(12):e0189338. doi: 10.1371/journal.pone.0189338 (PMC5726718; doi:10.1371/journal.pone.0189338)
Supplement: S3 Table — (PDF) [file pone.0189338.s008.pdf]

S3 Table**.** Gene names and Uniprot accession numbers of vertebrate and invertebrate Abl homologs used for multiple sequence alignment.

**Species**	**Gene name**	**Uniprot accession #**
Drosophila melanogaster (Fruit fly)	*Abl*	M9PFS1
Anopheles gambiae (African malaria mosquito)	*AgaP_AGAP004989*	A7UTI5
Apis mellifera (Honeybee)	*LOC409127*	A0A088AT87
Tribolium castaneum (Red flour beetle)	*TcasGA2_TC034634*	A0A139WK02
Heliconius melpomene (Postman butterfly)	*HMEL015066*	none - UniParc ID UPI000276D952
Zootermopsis nevadensis (Dampwood termite)	*L798_04869*	A0A067RM58
Pediculus humanus subsp. corporis (Body louse)	*PHUM538010*	E0VZT3
Tetranychus urticae (Two-spotted spider mite)	*LOC107359049*	T1JZW2
Stegodyphus mimosarum (African social velvet spider)	*X975_10925*	A0A087TJH9
Stegodyphus mimosarum (African social velvet spider)	*X975_14532*	A0A087T7S4
Caenorhabditis elegans	*abl-1*	P03949
Brugia malayi (Filarial nematode worm)	*Bma-abl-1*	A0A1P6C3H3
Helobdella robusta (Californian leech)	*HELRODRAFT_166124*	T1EXT6
Schistosoma mansoni (Blood fluke)	*Smp_169230*	G4VGW8
Strongylocentrotus purpuratus (Purple sea urchin)	*SP-ABL*	W4Z732
Octopus bimaculoides (California two-spotted octopus)	*OCBIM_22014525mg*	A0A0L8IDM9
Daphnia pulex (Water flea)	*DAPPUDRAFT_5206*	E9GB51
Mus musculus (Mouse)	*Abl1*	Q3SYK5
Mus musculus (Mouse)	*Abl2*	Q4JIM5
Monodelphis domestica (Gray short-tailed opossum)	*ENSMODG00000012126*	F7FU48
Monodelphis domestica (Gray short-tailed opossum)	*ABL2*	F7FED8
Homo sapiens (Human)	*ABL1*	P00519
Homo sapiens (Human)	*ABL2*	P42684
Ornithorhynchus anatinus (Duckbill platypus)	*ABL1*	F6QCG8
Ornithorhynchus anatinus (Duckbill platypus)	*ABL2*	F6YHH1
Pan troglodytes (Chimpanzee)	*ABL1*	H2QY21
Pan troglodytes (Chimpanzee)	*ABL2*	H2R8D7
Pongo abelii (Sumatran orangutan)	*ABL1*	H2PU80
Pongo abelii (Sumatran orangutan)	*ABL2*	H2N4J6
Gallus gallus (Chicken)	*ABL1*	F1NCD9
Gallus gallus (Chicken)	*ABL2*	A0A1D5PIY0
Gallus gallus (Chicken)	*ENSGALG00000042806*	A0A1D5P5Q8
Anolis carolinensis (Green anole)	*ABL1*	H9GNS0
Anolis carolinensis (Green anole)	*ABL2*	H9GIP7
Xenopus tropicalis (Western clawed frog)	*abl1*	F7BN18
Xenopus tropicalis (Western clawed frog)	*abl2*	F7BKF3
Danio rerio (Zebrafish)	*abl1*	F8W2F9
Danio rerio (Zebrafish)	*abl2*	B0UXN7
Gasterosteus aculeatus (Three-spined stickleback)	*abl1*	G3PX44
Gasterosteus aculeatus (Three-spined stickleback)	*abl2*	G3PAC1
