# Supplementary material for: Long disordered regions of the C-terminal domain of Abelson tyrosine kinase have specific and additive functions in regulation and axon localization
Source: PLoS One. 2017 Dec 12;12(12):e0189338. doi: 10.1371/journal.pone.0189338 (PMC5726718; doi:10.1371/journal.pone.0189338)
Supplement: S4 Table — (PDF) [file pone.0189338.s009.pdf]

S4 Table. Primers used in the generation of Abl mutant transgenes in this work.

Primer name	Sequence
1Q-F	CGACGGTACCAAAATG CATGCGCTGGAGCACATGTTT
1Q-R	GTGAGCGGCCGC GTCCATTCGTGCTGAGGTCGTC
2Q-F	AACAACCGGTCTCAAGGACGACCTCAGCACGAATGGAC
2Q-R	GTCTGCGGCCGCAA ATGGTGCCTTCGGTAGTGGTG
3Q-F	AACAACCGGTCTCAAGCAA ATGATGACGTTCTCCTC
3Q-R	GTGAGCGGCCGCGA GTGCCAACCGTATTGATGG
4Q-F	AAGTACCGGTCTCAAA ATCAATACGGTTGGCAC
4Q-R	AGTCGCGGCCGCC CCTGTTAAGCGCATTGGA
d1E-F	GGT CTCACGCCGAACGCCCAC
d1E-R	GGTCTCC TGCTTTTCCACCGCTTCGG
d2E-F	GGT CTCAAGGACGACCTCAGCACGAATGGAC
d2E-R	GGT GTTGTGGTGGGCGTTCGG
AblN-F	AGGCGCGCCAAA ATGGGGGCTCAGCAGGG
AblN-R	TGAGACCGGTCTCC TGCTTTTCCACCGCTTCGG
dC-R	ACATA CCGGAGAGCGCTGTGCAG
dPxxP-F	AAGCGAACCAGCCTGCTCTC
dPxxP-R	CGCCTGTTTGCCCTGCTTGT
dEVH1-F	CAGGCGCCGGAGAGCGCTG
dEVH1-R	CAGAGCTGGCTGGACTCC
dFABD-R	AGTCGCGGCCGCTCTGTGTGGA
